# Individual and socio-psychological characteristics as predictors of physical activity among men living with overweight and obesity taking part in the *Aussie Fans in Training* weight management behaviour change programme

**DOI:** 10.1177/00178969241300100

**Published:** 2024-11-23

**Authors:** Brendan J Smith, Joanne McVeigh, Dominika Kwasnicka, Hugh Riddell, Eleanor Quested

**Affiliations:** a Physical Activity and Well-Being Research Group, enAble Institute, Curtin University, Perth, WA, Australia; b Curtin School of Population Health, Curtin University, Perth, WA, Australia; c Curtin School of Allied Health, Curtin University, Perth, WA, Australia; d Movement Physiology Laboratory, School of Physiology, University of the Witwatersrand, Johannesburg, South Africa; e Melbourne School of Population and Global Health, The University of Melbourne, Melbourne, VIC, Australia

**Keywords:** Affective states, behaviour change, men’s health, obesity, physical activity

## Abstract

**Background:**

Sufficient physical activity (PA) is important to reduce the risk of men developing chronic diseases and to improve mental health. The effectiveness of PA programmes can vary, however, among men. Individual and socio-psychological characteristics may affect the level of men’s PA before starting a behaviour change programme as well as changes in PA during the programme.

**Aims:**

The primary objective of this study was to examine the role of individual and socio-psychological characteristics in predicting men’s (a) accelerometer-assessed PA (step count and moderate to vigorous PA [MVPA]) upon presentation to a behaviour change programme and (b) changes in PA (step count and MVPA) during a behaviour change programme.

**Methods:**

A total of 109 men (mean age = 45.5 years, *SD* = 7.8 years, mean body mass index = 34.5 kg/m^2^, *SD* = 5.1 kg/m^2^) who participated in the *Australian Fans in Training* 3-month PA and dietary behaviour change programme in 2018 participated in this study. Before and after completing the 12-week programme, men completed assessments including individual (age, weight) and socio-psychological (psychological needs support, psychological needs satisfaction, positive affect and negative affect) characteristics. We used regression analysis to examine the relationship between these variables and PA.

**Results:**

At baseline, men’s weight (β = –.36, *p* < .001) and positive affect (β = .29, *p* < .01) were significant predictors of step count. At baseline, men’s weight (β = –.21, *p* < .05) and negative affect (β = .23, *p* < .05) were significant predictors of MVPA, though the overall model did not display statistical significance. The overall regression models did not significantly predict changes in either step count or MVPA pre- to post-programme.

**Conclusion:**

The influence of weight and positive and negative affect in predicting PA outcomes suggests that tailoring men’s health behaviour change interventions to individual circumstances and needs could enhance their effectiveness for some men.

## Introduction

Insufficient physical activity (PA) in the form of not meeting the recommended guidelines of 150 minutes/week of moderate PA (Brown et al., 2013) has become a critical health concern (World Health Organization [WHO], 2022). Sufficient PA is associated with a reduction in risk of cardiovascular disease, type II diabetes and site-specific cancers (Bull et al., 2020). Sufficient PA can improve mental health, cognitive functioning and sleep, reduce the risk of falls and help maintain a healthy body weight (Bull et al., 2020). Conversely, insufficient PA is associated with increased mortality, with the relative risks associated with excessive or prolonged uninterrupted sedentary time reportedly higher among people who are insufficiently active (Lavie et al., 2019). Insufficient PA is also a risk factor for overweight and obesity, as measured by a body mass index (BMI) of >25 kg/m^2^ (Australian Institute of Health and Welfare [AIHW], 2023c). In Australia, it is estimated that 76% of Australians aged 18–64 years are considered insufficiently active (Australian Bureau of Statistics [ABS], 2021). Gender differences in the health risks of insufficient PA have been identified (e.g. Li and Siegrist, 2012). Men in Australia are twice as likely as women to be at risk for coronary heart disease (AIHW, 2023b), 1.4 times as likely to be at risk of stroke (AIHW, 2023b) and 1.3 times as likely to be at risk of type II diabetes (AIHW, 2023a). Men in Australia also have a higher incidence of overweight/obesity (75%) and cardiovascular disease (5%) than women (60% and 2%, respectively; ABS, 2018). In Australia, men have a lower life expectancy than women by 4.1 years (ABS, 2021). Increasing PA engagement among men is therefore a critical health concern.

Programmes and interventions tailored specifically to the interests, values and preferences of men can attract and engage men in PA (Sharp et al., 2020). Sports settings have been effectively used as a ‘hook’ to attract men to health interventions for weight management (George et al., 2022). For example, the *Football Fans in Training* (FFIT; Hunt et al., 2014) and, the Australian version of the same, *Australian Fans in Training* (Aussie-FIT; Kwasnicka et al., 2020) are interventions that take advantage of men’s interest in professional sports to encourage dietary and PA behaviour change. Both programmes were designed to support men in making small, sustainable changes to their eating and PA behaviour to improve their health. Each programme was delivered by a trained coach and consisted of 12 weekly, 90-minute face-to-face workshops, which included an educational component and supervised PA. Aussie-FIT coaches received additional training in motivationally supportive strategies.

A randomised controlled trial (RCT) of FFIT among 747 men in Scotland showed that the intervention group lost an average of 5.56 kg at 12 months, compared to a weight loss of 0.58 kg in the control group (Hunt et al., 2014). In addition, weight loss and dietary and PA improvements were maintained for 3.5 years after the start of the study, demonstrating the potential of this kind of programme for long-term behaviour change maintenance (Gray et al., 2018). FFIT was also adapted for wider utilisation in Europe (EuroFIT; Wyke et al., 2019) in a RCT across four countries (i.e. England, Netherlands, Norway and Portugal), with total PA (steps/day) and total sedentary time (minutes/day) as the primary outcomes. The EuroFIT programme proved effective in increasing PA (measured with accelerometry), with the intervention group completing significantly more steps/day than control participants. Both FFIT and EuroFIT were cost-effective and improved PA, weight, diet, health-related quality of life and well-being (Wyke et al., 2015, 2019).

That being said, FFIT, EuroFIT and the Aussie-FIT studies were limited in the extent to which they helped clarify the role of individual differences in predicting PA behaviour change. First, the primary outcome of FFIT was weight loss (Wyke et al., 2015), with PA as a secondary outcome assessed via self-report measures, which may not accurately reflect PA levels (Prince et al., 2020). Second, the Aussie-FIT study was not designed to explore the determinants of PA behaviour change due to its design as a pilot feasibility study (Quested et al., 2018). Over and above the primary and secondary objectives that have been explored in these behaviour change programmes, there is an opportunity to explore the individual characteristics (e.g. age, weight) and social-psychological factors (e.g. psychological need satisfaction, affect) that may influence PA behaviour change.

Older adults face a various number of barriers that can affect their participation in PA. Alongside barriers typically experienced by the general population (e.g. time constraints and bad weather; Aaltonen et al., 2012), older adults may also experience limited functional capacity (e.g. strength, mobility), perceived risk of injury (e.g. falls) and negative physical sensations such as pain (Meredith et al., 2023). Ageing has been shown to be associated with insufficient PA (Achttien et al., 2020), and age has been found to predict both the initiation and maintenance of PA (Burton et al., 1999). Overweight and obesity have been identified as associated with insufficient PA (Silveira et al., 2022) and there is evidence to suggest that physical inactivity can predict future weight gains (Pietiläinen et al., 2008). Importantly, people living with overweight and obesity also face barriers that can inhibit their participation in PA such as health problems, weight perception and low mood (McIntosh et al., 2016). Yet, there have been limited studies exploring whether weight status can predict future engagement in PA.

In addition to the individual characteristics that have been largely unexplored in men’s PA interventions, socio-psychological characteristics such as motivation (Rahman et al., 2011) and affect (Forster et al., 2021) are yet to be investigated. Quality of motivation is known to be a critical determinant of sustained engagement in PA (Wang and Hagger, 2023). Self-determination theory (SDT; Deci and Ryan, 1985) predicts that motivationally adaptive social environments can foster better quality of motivation, and in turn positive cognitive, behavioural and affective outcomes. From an SDT perspective, needs support includes support for basic psychological needs for autonomy, competence and relatedness. This can be achieved by using autonomy-supportive language, providing optimal challenges that are not overwhelming and creating a supportive and collaborative group environment (Deci and Ryan, 2000). Need support in the form of need supportive behaviour from others can be effective in helping people engage in PA (Wang and Hagger, 2023). Satisfaction of the need for autonomy (i.e. having a sense of control and choice in decision-making), competence (i.e. feeling capable of doing a task) and relatedness (i.e. having a sense of belonging and inclusion) have been found to predict engagement in PA (Reifsteck et al., 2023). These feelings are best conceptualised in SDT as basic psychological needs, and the extent to which an individual feels these needs are met is considered psychological needs satisfaction.

With regard to Aussie-FIT, the level of psychological needs satisfaction may predict how active men were before and after participating in the programme. This is of particular interest given that the Aussie-FIT programme was designed to increase feelings of autonomy, competence and relatedness. In addition, need supportive significant others (e.g. a spouse and family) outside of the intervention context may influence PA levels and are also important to consider when evaluating factors that might influence the outcomes of a programme.

People experience various emotions and moods (e.g. tension and satisfaction), best conceptualised as positive or negative affect (Watson and Tellegen, 1985). While the effects of PA on individual affect are widely acknowledged (Emerson and Williams, 2015), there is less clarity on how affect influences PA. Results from a recent systematic review suggest that affective states at one time may predict future PA engagement (Forster et al., 2021). One study identified in the review with a sample of mostly men (70%) found significantly more patients in a positive affect intervention group increased PA energy expenditure by 336 kcal/week compared to a control group (Peterson et al., 2012). Another study with a sample of people living with obesity found that participants were more likely to exercise on days when they had experienced incidental positive affect earlier in the day (Emerson et al., 2018). The review concludes, however, that further research is required to test the complex relationship, hinting at the potential predictive role that affect may have in determining men’s PA levels.

## The present study

In sum, in the case of men’s health interventions delivered in sports clubs, there has been limited exploration of the socio-psychological and individual characteristics that may predict how active one is when enrolling in a programme. Acknowledging the dynamic and evolving relationship between individual and socio-psychological characteristics and PA levels, it would also be beneficial to investigate how changes in these characteristics from pre- to post-programme might predict changes in PA levels to better understand potential predictors of PA for men participating in the Aussie-FIT programme. If significant predictors are identified, future programmes might consider screening men entering programmes to identify characteristics that could influence their likelihood of engaging in PA, and initiating early supplementary interventions tailored to provide additional support based on the psychological and individual characteristics of the programme participants. Variables predictive of adaptive change in PA could be considered in future developments of men’s PA interventions.

Against this background, this study aims to explore the influence of individual and socio-psychological characteristics on PA, among men participating in Aussie-FIT. The individual characteristics investigated were age and weight and socio-psychological characteristics were need support from significant others, need satisfaction and positive and negative affect. PA was measured in terms of step count and moderate to vigorous PA (MVPA). This study proposed the following hypotheses.

1. Men’s individual (age and weight) and socio-psychological (need support from significant others, need satisfaction, positive and negative affect) characteristics will predict PA levels at the start of the programme.
2. The same characteristics will predict changes in PA from pre- to post-programme.
3. Changes in men’s weight, need support from significant others, need satisfaction and positive and negative affect will predict changes in PA from pre- to post-programme.

## Method

### Participants

Inclusion criteria were men aged 35–65 years with a BMI ⩾28 kg/m^2^. Of the 130 men who completed the Aussie-FIT programme in 2018 (Kwasnicka et al., 2020), data collected from 109 men (mean age = 45.8 years, *SD* = 7.9 years, mean BMI = 34.5 kg/m^2^, *SD* = 5.1 kg/m^2^) who had completed pre-programme PA assessments were used in the study. Of the included men, 94% identified their ethnic origin as Caucasian, 4% as Mixed and 2% as Other. The average years of full-time education was 14 years (*SD* = 3.16 years). Approximately, 95% of the men were in paid employment or self-employed, and they worked an average of 41 hours/week.

### Procedure

Permission to conduct this study was obtained from the Curtin University Human Research Ethics Committee (Reference: HRE2017-0458). Upon commencement of the Aussie-FIT pilot trial, men provided consent for their de-identified data to be analysed once follow-up measures had concluded. The Aussie-FIT study was designed as an RCT with a waitlist control group. Data were pooled from pre- and post-intervention measures taken before and after the intervention. Participants in both groups completed measures approximately 1 week before the commencement of the programme and approximately 1 week following the conclusion of the programme. Data were collected by trained researchers at the football clubs where the programme took place. Men were provided with AUD$20 gift cards at each measurement session to thank them for their attendance. The full feasibility study protocol can be found in Quested et al.’s (2018) study.

### Measures

#### Age and weight

A self-report questionnaire was used to record the participant’s age. Weight in kilogrammes was measured using a valid and reliable electronic scale (SECA 813 Flat Scale, EMSE81; Birmingham, UK) at each assessment time point.

#### Physical activity

Participants were asked to wear an Actigraph GTX-9 (Actigraph LLC, Pensacola, FL, USA) accelerometer device on the hip for 8 days. The devices were programmed to record data at a frequency of 30 Hz, and data were collapsed into 60-second epochs. Accelerometer data were downloaded and processed in SAS (version 9.3, SAS Institute, Cary, NC, USA), using a validated semi-automated algorithm (McVeigh et al., 2016). During the processing, the algorithm separated waking wear PA data from non-wear or sleep data. On completion of the intervention, men repeated the accelerometer wear protocol. Common cut-points were used to classify each minute as sedentary (<100 counts/minute [cpm]), light intensity (100–1,951 cpm), moderate intensity (1,952–5,724 cpm) or vigorous intensity (>5,724 cpm; Freedson et al., 1998; Matthews et al., 2008). All participants with ⩾4 valid days (i.e. ⩾10 hours of waking wear time) of data were included in the analyses. The measures of PA extracted from the accelerometer data that were used in the analyses were minutes of PA intensity (i.e. minutes spent in MVPA) and step count (McVeigh et al., 2016 ). The average waking wear time (minutes/day) for each participant was also obtained and included in all analyses as a covariate.

#### Psychological need support

The 12-item Interpersonal Behaviours Questionnaire was used to assess men’s perceptions of psychological need support from others in life in relation to weight loss (Rocchi et al., 2017). Participants were presented with the statement, ‘When doing things relevant to achieving or maintaining a healthy body weight, I felt that others in my life . . .’ followed by 12 reasons (e.g. ‘. . . told me I can accomplish things’ and ‘. . . supported my decisions’). Responses were measured on 7-point Likert-type scales ranging from 1 (‘Do not agree at all’) to 7 (‘Completely agree’). Scores from each item were collapsed into one overall score, with a higher score indicating greater perceived need support.

#### Psychological need satisfaction

Basic psychological need satisfaction in relation to weight loss was measured with the autonomy and competence sub-scales from the Treatment Self-Regulation Questionnaire of weight loss motivation by Chen et al. (2015) and four items tapping relatedness satisfaction (Richer and Vallerand, 1998). Participants were presented with the statement, ‘When doing things relevant to achieving or maintaining a healthy body weight, I felt . . .’ followed by 12 reasons tapping autonomy (four items, e.g. ‘. . . a sense of choice and freedom in what I do’), competence (four items, e.g. ‘. . . competent to achieve my goals’) and relatedness (four items, e.g. ‘. . . supported’). Responses were measured on 5-point Likert-type scales ranging from 1 (‘strongly disagree’) to 5 (‘strongly agree’). Scores for each need were collapsed for the analyses, where higher scores were indicative of higher need satisfaction.

#### Positive and negative affect

Positive and negative affect was measured using the short form, 10-item, version of the Positive and Negative Affect Scale (Thompson, 2007). Participants were presented with the statement, ‘During [the] last month, I generally felt . . .’ followed by five negative affect indicators (e.g. ‘upset’) and five positive affect indicators (e.g. ‘inspired’). Responses were measured on 5-point Likert-type scales ranging from 1 (‘Not at all’) to 5 (‘Extremely’). The two constructs were separated, and each construct was totalled and divided by the number of items to provide an average score. Higher scores indicate higher positive or negative affect.

### Statistical analysis

Hierarchical linear regression models were used to examine the role of individual (i.e. age and weight) and socio-psychological (i.e. need support, need satisfaction and positive and negative affect) characteristics in relation to PA data (steps and MVPA) pre-programme and pre-to-post individual changes. Prior to analysis, assumptions of normality, linearity and homoscedasticity were examined, in addition to evaluating collinearity statistics (i.e. tolerance and Variance Inflation Factor). No assumptions were violated and thus a standard, parametric linear regression (e.g. Cohen et al., 2002) was deemed appropriate. The analyses were conducted using SPSS 28.0 software.

To assess whether pre-programme values of the target variables predicted PA at the start of the programme, pre-intervention MPVA (model 1) and step count (model 2) were included as dependent variables in separate analyses. The predictor variables were entered into the models in five blocks. Average accelerometer waking wear time (minutes/day) was entered into block 1 to control for variability in wear time between participants. Age and weight were included in block 2. Need support (block 3), need satisfaction (block 4) and positive and negative affect (block 5) at pre-programme were added as predictors.

To assess subsequent hypotheses, post-intervention MPVA (model 3) and step count (model 4) were included as dependent variables in separate analyses. To assess whether pre-programme values of the target variables predicted change in PA from pre- to post-programme, the same selection and order of predictors as above were taken, except pre-programme MVPA and step count were included in block 1 so that the analyses would represent predictors of change from baseline to follow-up. The third hypotheses focused on whether changes in the target predictors predicted changes in PA. Accordingly, pre-programme and post-programme average accelerometer wear time (minutes/day) and pre-programme PA, weight, need support, need satisfaction and positive and negative affect were included in the first step to control for the influence of these characteristics when assessing change in PA. Post-programme need support (block 3), post-programme need satisfaction (block 4) and post-programme positive and negative affect (block 5) were subsequently added as predictors. The coefficients, standard errors and standardised coefficients for each predictor variable in each block were reported, as well as the *R*-squared value and change in the *R*-squared value for each model. The significance level was set at .05. The magnitude of standardised correlations between the dependent and independent variables was interpreted as follows: ‘weak’ (<0.02–0.13), ‘moderate’ (0.13–0.26) and ‘substantial’ (⩾0.26) effect sizes (Cohen, 1988).

## Results

Both dependent variables (step count and MVPA) displayed a mean increase from pre- to post-programme. Further descriptive statistics of relevant study variables are reported in Table 1. Bivariate correlations, which represent an important effect size measure for regression analyses (Hoyt et al., 2008), are reported in the Supplemental Material (S1).

**Table 1.** Descriptive statistics for study variables.

Variable	Pre-programme			Post-programme		
	Cronbach’s alpha	*M* (*SD*)	Range	Cronbach’s alpha	*M* (*SD*)	Range
Individual characteristics						
_Age (years)		45.45 (7.82)	35–65			
_Weight (kg)		109.27 (17.16)	75–190		106.49 (17.04)	72–182
Socio-psychological characteristics						
_Need support	.908	5.21 (1.18)	2–7	.929	5.5 (1.22)	2–7
_Need satisfaction	.951	3.35 (0.63)	2–5	.972	3.94 (0.65)	2–5
_Positive affect	.817	2.94 (0.67)	1–5	.811	3.52 (0.63)	2–5
_Negative affect	.738	1.71 (0.62)	1–4	.804	1.51 (0.56)	1–4
Physical activity (accelerometer measures)						
_Waking wear time (minutes/day)		922 (136)	664–1,329		913 (167)	642–1,361
_MVPA (minutes/day)		36.58 (19.93)	8–103		45.32 (21.59)	11–103
_Daily step count		13,134 (3,896)	5,812–25,205		14,381 (4,305)	6,638–25,524

Table 2 presents the results from the two hierarchical regression models. The fifth step of model 1 which considers the combined effects of all predictors on step count pre-programme was significant *F*(7, 100) = 4.53, *p* < .05, accounting for a moderate proportion of variability in step count pre-programme *R*^2^ = .24. The unstandardised coefficient for weight indicated that every additional kilogramme in body weight was associated with 83 fewer steps/day (*b* = –82.8, 95% CI [–124.5, –41.1]). The unstandardised coefficient for positive affect indicated that every additional point in positive affect was associated with 1,709 more steps/day (*b* = 1,709, 95% CI [555, 2,862]). There were no significant individual regression coefficients between the remaining characteristics (age, need support, need satisfaction and negative affect) and step count pre-programme.

**Table 2.** Hierarchical regression results examining relationships between physical activity and individual and socio-psychological characteristics at baseline.

Outcomes (*n* = 109)	Model 1 – step count					Model 2 – MVPA				
*Predictors*	*B* (*SE*)	95% CI	β	*R* ^2^	Δ*R*^2^	*B* (*SE*)	95% CI	β	*R* ^2^	Δ*R*^2^
Step 1				.018	.018				<.001	<.001
Waking wear time (minutes/day)	3.83 (2.76)	–1.63, 9.29	.134			.002 (.014)	–.026, .03	.012		
Step 2				.147***	.13				.037	.037
Waking wear time (minutes/day)	2.01 (2.65)	–3.25, 7.26	.07			–.003 (.014)	–.031, .026	–.018		
Weight (kg)	–84 (21.41)	–127.45, –42.55	–.373***			–.23 (.116)	–.46, .0003	–.198		
Age (years)	–26.34 (46.28)	–118.12, 65.45	–.053			–.159 (.251)	–.656, .339	–.063		
Step 3				.157	.01				.047	.010
Waking wear time (minutes/day)	1.68 (2.66)	–3.6, 6.96	.059			–.004 (.014)	–.033, .024	–.029		
Weight (kg)	–84.06 (21.4)	–126.5, –41.62	–.37***			–.226 (.116)	–.456, .005	–.194		
Age (years)	–21.03 (46.48)	–113.22, 71.16	–.042			–.132 (.252)	–.632, .368	–.052		
Need support	334.15 (302.39)	–265.56, 933.87	.101			1.67 (1.64)	–1.58, 4.93	.099		
Step 4				.164	.007				.062	.015
Waking wear time (minutes/day)	1.85 (2.67)	–3.45, 7.15	.065			–.003 (.014)	–.032, .026	–.02		
Weight (kg)	–83.42 (21.43)	–125.93, –40.91	–.366***			–.221 (.116)	–.45, .009	–.19		
Age (years)	–30.89 (47.81)	–125.74, 63.94	–.062			–.207 (.258)	–.719, .305	–.082		
Need support	146.85 (367.69)	–582.47, 876.17	.044			.247 (1.99)	–3.69, 4.19	.015		
Need satisfaction	619.21 (690.19)	–749.78, 1,988.2	.1			4.72 (3.73)	–2.68, 12.11	.149		
Step 5				.241**	.077				.124	.063
Waking wear time (minutes/day)	1.94 (2.57)	–3.17, 7.04	.068			–.003 (.014)	–.031, .025	–.021		
Weight (kg)	–82.81 (21.02)	–124.51, –41.11	–.363***			–.246 (.115)	–.474, –.018	–.212*		
Age (years)	–32.54 (46.03)	–123.87, 58.79	–.065			–.208 (.252)	–.708, .292	–.082		
Need support	175.2 (354.22)	–527.57, 877	.053			.437 (1.94)	–3.41, 4.28	.026		
Need satisfaction	50.27 (740.4)	–1,418.65, 1,519.19	.008			4.29 (4.05)	–3.75, 12.33	.136		
Positive affect	1,709.04 (581.57)	555.22, 2,862.86	.294**			5.43 (3.18)	–.88, 11.75	.184		
Negative affect	923.05 (602.55)	–272.4, 2,118.5	.146			7.39 (3.3)	.85, 13.94	.23*		

* *p* < .05. **p < .01. ***p < .001.

The fifth step of model 2 which considers the combined effects of all predictors on MVPA pre-programme was not significant, *F*(7,100) = 2.03, *p* = .059, with the coefficient of determination indicating these variables explained a small proportion of variation in the dependent variable (*R*^2^ = .12). The unstandardised coefficient for weight indicated that every additional kilogramme in body weight was associated with 15 fewer seconds of MVPA/day (*b* = –.25, 95% CI [–.47, –.02]). The unstandardised coefficient for negative affect indicated that every additional point in negative affect was associated with 7.4 more minutes of MVPA/day (*b* = 7.39, 95% CI [.85, 13.94]). There were no significant individual regression coefficients between the remaining characteristics (age, need satisfaction, need support and positive affect) and MVPA pre-programme. However, given the non-significance of the overall model, results should be interpreted with caution.

There were 45 missing cases of PA data post-programme. Therefore, while the sample size in the analyses for models 1 and 2 was *n* = 109, subsequent models were limited to a sample of *n* = 64. The overall regression models did not significantly predict changes in either step count or MVPA pre- to post-programme after controlling for the variables entered in step 1 of the hierarchical regressions. We provide the results of these models in the Supplemental Material (S2).

Given the substantial number of participants with missing data (step count and MVPA), we ran additional exploratory regression models using a Markov Chain Monte Carlo method for multiple imputation to substitute missing instances, enabling us to include all cases in the analysis (*n* = 109). The overall regression model was a significant predictor of change in step count, *F*(7, 100) = 13.45, *p* < .05, accounting for approximately half of the variance of change in step count from pre- to post-programme (*R*^2^ = .52). Similarly, the overall regression model was a significant predictor of change in MVPA, *F*(7,100) = 4.42, *p* < .05, with the coefficient of determination indicating that the independent variables account for a moderate proportion of variation in the dependent variable (*R*^2^ = .26). None of the changes in participant characteristics were significant predictors of changes in step count or MVPA. These results should be interpreted with caution; however, considering 41% of the PA data post-programme was subject to multiple imputation. The full results of these analyses are available from the corresponding author upon request.

## Discussion

This study explored the predictive relationship between individual (age and weight) and socio-psychological (psychological need support, psychological need satisfaction and positive and negative affect) characteristics on accelerometer-assessed PA (step count and MVPA). A series of six hierarchical multiple linear regression analyses were conducted to better understand potential predictors of PA for men participating in the Aussie-FIT programme. Overall, the findings from this study partially support the hypotheses, highlighting the role that weight and affect (positive and negative) have on men’s engagement in PA before commencing a PA behaviour change programme.

The results indicate that there was a negative relationship between weight and PA upon commencing a PA behaviour change programme, such that men with a higher weight completed fewer steps and fewer minutes of MVPA/day than men with a lower weight. In addition, affect appeared to play a role in men’s level of PA upon commencing a PA behaviour change programme. There was a significant positive relationship between positive affect and step count, such that men with higher positive affect completed more steps/day than men with a lower positive affect. There was also a significant positive relationship between negative affect and MVPA, such that men with higher negative affect completed more minutes of MVPA than men with lower negative affect.

With regard to weight, these results are consistent with the existing literature suggesting weight status plays a role in PA behaviour (e.g. Pietiläinen et al., 2008; Silveira et al., 2022). A recent meta-analysis found 45% of 111,815 individuals living with obesity were insufficiently active (Silveira et al., 2022). The BMI cut-off for inclusion in this meta-analysis was >30 kg/m^2^ which is akin to the mean BMI of the sample in this study of 34.5 kg/m^2^, *SD* = 5.1 kg/m^2^. These results confirm the relationship between weight status and PA and recognise the predictive nature of weight status on PA. Because of this, it is important to recognise that the success of FFIT-styled interventions in changing PA may vary, depending on participants’ weight status.

It is evident that positive and negative affect both play a role in engagement in PA. Interestingly, however, the results from this study demonstrate that the relationship appears to differ depending on the use of different PA metrics. Previous research indicates that those with greater positive affect and lesser negative affect are more likely to engage in PA (Forster et al., 2021). In this study, the findings regarding the relationship between affect and MVPA are inconsistent with the literature, suggesting that on presentation to a PA programme, those with greater negative affect complete more minutes of MVPA/day than those with lesser negative affect. This was an unexpected finding and contrary to the hypotheses that those with greater negative affect would engage in less MVPA. Further research is required to understand the mechanisms behind this relationship.

Given the different conclusions between MVPA and step count metrics, this study highlights the importance of considering PA intensity in research design and methodology. During a period of PA, the volume of steps may be high but at a slower pace (i.e. less intense), whereas MVPA requires a greater acceleration (i.e. more intense activity). PA intensity is an important consideration for health outcomes. For instance, high-intensity PA has been shown to be associated with improved outcomes for people with lower back pain (Ram et al., 2023) and may protect against symptoms of anxiety and depression (Shannon et al., 2023). Conversely, low-intensity PA has been demonstrated to improve flexibility, balance and lower limb muscle strength, and reduce depressive symptoms among older adults (Tse et al., 2015). One study has also found differences in health outcomes between minutes of MVPA and step count, indicating that a 150-minutes/week MVPA threshold leads to greater reductions in BMI and blood sugar levels (A1C) than a 10,000 steps/day threshold (Hajna et al., 2018). Our study also suggests that there may be different individual and socio-psychological predictors of how much someone moves, compared to predictors of how intensely someone moves. The metric of PA measurement in future PA trials therefore requires careful consideration as it may influence the conclusions that can be drawn.

Although PA accelerometry is often viewed as an objective measure, accelerometry data are not free of researcher influence and measurement error. Results from this study highlight how the choice of outcome measure when measuring PA with accelerometers could lead to different conclusions. For example, weight and positive affect were predictive of step count, but not related to MVPA in the present study. Thus, while both step count and MVPA have health benefits, future programmes should carefully consider which outcomes they want to improve.

### Limitations

A key limitation of this study was the amount of missing PA data post-intervention. While results from the all-cases analyses, which used multiple imputation to account for missing data, showed the same pattern of results as the main analyses, future predictive studies akin to this study would benefit from ways to retain participants for the post-intervention measures.

Socioeconomic factors (e.g. place of residence, income, occupation and educational attainment) may also influence engagement in PA. For instance, individuals with higher levels of education may have a greater understanding of the benefits of PA and better access to resources and opportunities for PA than their counterparts with lower levels of education (Nutbeam and Lloyd, 2021). A recent systematic review examining how weight management studies consider men’s socioeconomic status highlights the need for interventions designed to appeal to men across different socioeconomic groups (Mcdonald et al., 2022). Socioeconomic factors may play a role in health behaviours at the beginning of PA interventions such as Aussie-FIT and the extent to which PA was initiated and maintained. While data on ethnic background, years of education and employment status were collected in this study, they were not included in the analyses as we needed to maintain statistical power and reduce the theoretical complexity of the models. This decision was made to limit the number of tested predictors, prioritising those deemed to be more theoretically relevant during the study’s design phase.

It is also important to acknowledge the limited generalisability of findings from this study. The sample was not representative of the Australian population, and consisted of mostly white, well-educated and employed individuals.

## Conclusion

Findings from this study shed light on the unique challenges faced in men’s health promotion. Results provide valuable insights into the characteristics contributing to PA behaviour. Specifically, weight and positive and negative affect appear to play a role in how active men are on presentation to a PA programme. This is important to know because understanding the factors influencing PA behaviour in men can help pave the way for the design and testing of individually tailored approaches that could supplement group interventions such as Aussie-FIT. For instance, additional functional and psychological support could be provided to men living with a higher body weight to help them overcome PA barriers such as pain and fear. In addition, those who present with lesser positive affect may benefit from tailored messaging to reduce tension or quell social expectations. Findings from this study may be useful for intervention designers, healthcare and clinical providers, academic and research institutions and community and patient organisations.

## Supplemental Material

sj-docx-1-hej-10.1177_00178969241300100 – Supplemental material for Individual and socio-psychological characteristics as predictors of physical activity among men living with overweight and obesity taking part in the Aussie Fans in Training weight management behaviour change programme Supplemental material, sj-docx-1-hej-10.1177_00178969241300100 for Individual and socio-psychological characteristics as predictors of physical activity among men living with overweight and obesity taking part in the Aussie Fans in Training weight management behaviour change programme by Brendan J Smith, Joanne McVeigh, Dominika Kwasnicka, Hugh Riddell and Eleanor Quested in Health Education Journal

sj-docx-2-hej-10.1177_00178969241300100 – Supplemental material for Individual and socio-psychological characteristics as predictors of physical activity among men living with overweight and obesity taking part in the Aussie Fans in Training weight management behaviour change programme Supplemental material, sj-docx-2-hej-10.1177_00178969241300100 for Individual and socio-psychological characteristics as predictors of physical activity among men living with overweight and obesity taking part in the Aussie Fans in Training weight management behaviour change programme by Brendan J Smith, Joanne McVeigh, Dominika Kwasnicka, Hugh Riddell and Eleanor Quested in Health Education Journal

## References

[bibr1-00178969241300100] Aaltonen S Leskinen T Morris T , et al. (2012) Motives for and barriers to physical activity in twin pairs discordant for leisure time physical activity for 30 years. International Journal of Sports Medicine 33(2): 157–163. 22318531 10.1055/s-0031-1287848

[bibr2-00178969241300100] Achttien RJ van Lieshout J Wensing M , et al. (2020) The decline in physical activity in aging people is not modified by gender or the presence of cardiovascular disease. European Journal of Public Health 30(2): 333–339. 31562513 10.1093/eurpub/ckz159 PMC7183365

[bibr3-00178969241300100] Australian Bureau of Statistics (ABS) (2018) Overweight and obesity. Available at: www.abs.gov.au/statistics/health/health-conditions-and-risks/overweight-and-obesity/2017-18 (accessed 12 September 2023).

[bibr4-00178969241300100] Australian Bureau of Statistics (ABS) (2021) Physical activity. Available at: www.abs.gov.au/statistics/health/health-conditions-and-risks/physical-activity/latest-release (accessed 12 September 2023).

[bibr5-00178969241300100] Australian Institute of Health and Welfare (AIHW) (2023a) Diabetes. Available at: www.aihw.gov.au/reports-data/health-conditions-disability-deaths/diabetes/overview (accessed 12 September 2023).

[bibr6-00178969241300100] Australian Institute of Health and Welfare (AIHW) (2023b) Heart, stroke & vascular diseases. Available at: www.aihw.gov.au/reports/heart-stroke-vascular-diseases/hsvd-facts/contents/about (accessed 12 September 2023).

[bibr7-00178969241300100] Australian Institute of Health and Welfare (AIHW) (2023b) Risk factors to health. Available at: www.aihw.gov.au/reports/risk-factors/risk-factors-to-health/contents/overweight-and-obesity (accessed 12 September 2023).

[bibr8-00178969241300100] Brown WJ Bauman AE Bull F , et al. (2013) Development of evidence-based physical activity recommendations for adults (18-64 years). Report, Australian Government Department of Health, August.

[bibr9-00178969241300100] Bull FC Al-Ansari SS Biddle S , et al. (2020). World Health Organization 2020 guidelines on physical activity and sedentary behaviour. British Journal of Sports Medicine 54(24): 1451–1462. 33239350 10.1136/bjsports-2020-102955 PMC7719906

[bibr10-00178969241300100] Burton LC Shapiro S German PS (1999) Determinants of physical activity initiation and maintenance among community-dwelling older persons. Preventive Medicine 29(5): 422–430. 10564634 10.1006/pmed.1999.0561

[bibr11-00178969241300100] Chen B Vansteenkiste M Beyers W , et al. (2015) Basic psychological need satisfaction, need frustration, and need strength across four cultures. Motivation and Emotion 39(2): 216–236.

[bibr12-00178969241300100] Cohen J (1988) Statistical Power Analysis for the Behavioral Sciences. 2nd ed. New York: Routledge.

[bibr13-00178969241300100] Cohen J Cohen P West SG , et al. (2002) Applied Multiple Regression/Correlation Analysis for the Behavioral Sciences. 3rd ed. New York: Routledge.

[bibr14-00178969241300100] Deci EL Ryan RM (1985) Intrinsic Motivation and Self-Determination in Human Behavior. New York: Plenum.

[bibr15-00178969241300100] Deci EL Ryan RM (2000) The ‘what’ and ‘why’ of goal pursuits: Human needs and the self-determination of behavior. Psychological Inquiry 11(4): 227–268.

[bibr16-00178969241300100] Emerson JA Williams DM (2015) The multifaceted relationship between physical activity and affect. Social and Personality Psychology Compass 9(8): 419–433.

[bibr17-00178969241300100] Emerson JA Dunsiger S Williams DM (2018) Reciprocal within-day associations between incidental affect and exercise: An EMA study. Psychology & Health 33(1): 130–143. 28665227 10.1080/08870446.2017.1341515 PMC5738286

[bibr18-00178969241300100] Forster AK Richards EA Foli KJ , et al. (2021) Influence of affect on physical activity: An integrative review. Clinical Nursing Research 30(7): 934–949. 33111569 10.1177/1054773820968039

[bibr19-00178969241300100] Freedson PS Melanson E Sirard J (1998) Calibration of the Computer Science and Applications, Inc. accelerometer. Medicine and Science in Sports and Exercise 30(5): 777–781. 9588623 10.1097/00005768-199805000-00021

[bibr20-00178969241300100] George ES El Masri A Kwasnicka D , et al. (2022) Effectiveness of adult health promotion interventions delivered through professional sport: Systematic review and meta-analysis. Sports Medicine 52(11): 2637–2655. 35708886 10.1007/s40279-022-01705-z PMC9585012

[bibr21-00178969241300100] Gray CM Wyke S Zhang R , et al. (2018) Long-term weight loss trajectories following participation in a randomised controlled trial of a weight management programme for men delivered through professional football clubs: A longitudinal cohort study and economic evaluation. International Journal of Behavioral Nutrition and Physical Activity 15(1): 60. 29954449 10.1186/s12966-018-0683-3 PMC6022303

[bibr22-00178969241300100] Hajna S Ross NA Dasgupta K (2018) Steps, moderate-to-vigorous physical activity, and cardiometabolic profiles. Preventive Medicine 107: 69–74. 29126915 10.1016/j.ypmed.2017.11.007 PMC6625960

[bibr23-00178969241300100] Hoyt WT Imel ZE Chan F (2008) Multiple regression and correlation techniques: Recent controversies and best practices. Rehabilitation Psychology 53(3): 321–339.

[bibr24-00178969241300100] Hunt K Wyke S Gray CM , et al. (2014) A gender-sensitised weight loss and healthy living programme for overweight and obese men delivered by Scottish Premier League football clubs (FFIT): A pragmatic randomised controlled trial. The Lancet 383(9924): 1211–1221. 10.1016/S0140-6736(13)62420-4 PMC4524002 24457205

[bibr25-00178969241300100] Kwasnicka D Ntoumanis N Hunt K , et al. (2020) A gender-sensitised weight-loss and healthy living program for men with overweight and obesity in Australian Football League settings (Aussie-FIT): A pilot randomised controlled trial. PLoS Medicine 17(8): e1003136. 32760144 10.1371/journal.pmed.1003136 PMC7410214

[bibr26-00178969241300100] Lavie CJ Ozemek C Carbone S , et al. (2019) Sedentary behavior, exercise, and cardiovascular health. Circulation Research 124(5): 799–815. 30817262 10.1161/CIRCRESAHA.118.312669

[bibr27-00178969241300100] Li J Siegrist J (2012) Physical activity and risk of cardiovascular disease – A meta-analysis of prospective cohort studies. International Journal of Environmental Research and Public Health 9(2): 391–407. 22470299 10.3390/ijerph9020391 PMC3315253

[bibr28-00178969241300100] McDonald MD Hunt K Sivaramakrishnan H , et al. (2022) A systematic review examining socioeconomic factors in trials of interventions for men that report weight as an outcome. Obesity Reviews 23(7): e13436. 35187778 10.1111/obr.13436 PMC9285916

[bibr29-00178969241300100] McIntosh T Hunter DJ Royce S (2016) Barriers to physical activity in obese adults: A rapid evidence assessment. Journal of Research in Nursing 21(4): 271–287.

[bibr30-00178969241300100] McVeigh JA Winkler EA Healy GN , et al. (2016) Validity of an automated algorithm to identify waking and in-bed wear time in hip-worn accelerometer data collected with a 24 h wear protocol in young adults. Physiological Measurement 37(10): 1636–1652. 27652717 10.1088/0967-3334/37/10/1636

[bibr31-00178969241300100] Matthews CE Chen KY Freedson PS , et al. (2008) Amount of time spent in sedentary behaviors in the United States, 2003–2004. American Journal of Epidemiology 167(7): 875–881. 18303006 10.1093/aje/kwm390 PMC3527832

[bibr32-00178969241300100] Meredith SJ Cox NJ Ibrahim K , et al. (2023) Factors that influence older adults’ participation in physical activity: A systematic review of qualitative studies. Age and Ageing 52(8): afad145. 10.1093/ageing/afad145 PMC10438214 37595070

[bibr33-00178969241300100] Nutbeam D Lloyd JE (2021) Understanding and responding to health literacy as a social determinant of health. Annual Review of Public Health 42(1): 159–173. 10.1146/annurev-publhealth-090419-102529 33035427

[bibr34-00178969241300100] Peterson JC Charlson ME Hoffman Z , et al. (2012) A randomized controlled trial of positive-affect induction to promote physical activity after percutaneous coronary intervention. Archives of Internal Medicine 172(4): 329–336. 22269589 10.1001/archinternmed.2011.1311 PMC3717982

[bibr35-00178969241300100] Pietiläinen KH Kaprio J Borg P , et al. (2008) Physical inactivity and obesity: A vicious circle. Obesity 16(2): 409–414. 18239652 10.1038/oby.2007.72 PMC2249563

[bibr36-00178969241300100] Prince SA Cardilli L Reed JL , et al. (2020) A comparison of self-reported and device measured sedentary behaviour in adults: A systematic review and meta-analysis. International Journal of Behavioral Nutrition and Physical Activity 17(1): 31. 32131845 10.1186/s12966-020-00938-3 PMC7055033

[bibr37-00178969241300100] Quested E Kwasnicka D Thøgersen-Ntoumani C , et al. (2018) Protocol for a gender-sensitised weight loss and healthy living programme for overweight and obese men delivered in Australian football league settings (Aussie-FIT): A feasibility and pilot randomised controlled trial. BMJ Open 8(10): e022663. 10.1136/bmjopen-2018-022663 PMC6196804 30337315

[bibr38-00178969241300100] Rahman RJ Thogersen-Ntoumani C Thatcher J , et al. (2011) Changes in need satisfaction and motivation orientation as predictors of psychological and behavioural outcomes in exercise referral. Psychology & Health 26(11): 1521–1539. 22111661 10.1080/08870446.2010.538849

[bibr39-00178969241300100] Ram AK Summers SJ Booth J , et al. (2023) Higher intensity exercise reduces disability more than lower intensity exercise in adults with chronic low back pain: A systematic review and meta-analysis. Musculoskeletal Care 21(3): 611–622. 36647210 10.1002/msc.1734

[bibr40-00178969241300100] Reifsteck EJ Hevel DJ Anderson SN , et al. (2023) Investigating intraindividual variability of psychological needs satisfaction and relations with subsequent physical activity. Journal of Sport and Exercise Psychology 45(3): 166–170. 37160291 10.1123/jsep.2022-0140

[bibr41-00178969241300100] Richer SF Vallerand RJ (1998) Construction et validation de l’Échelle du sentiment d’appartenance sociale (ÉSAS). European Review of Applied Psychology 48(2): 129–138.

[bibr42-00178969241300100] Rocchi M Pelletier L Cheung S , et al. (2017) Assessing need-supportive and need-thwarting interpersonal behaviours: The Interpersonal Behaviours Questionnaire (IBQ). Personality and Individual Differences 104: 423–433.

[bibr43-00178969241300100] Shannon S Shevlin M Brick N , et al. (2023) Frequency, intensity and duration of muscle strengthening activity and associations with mental health. Journal of Affective Disorders 325: 41–47. 36587908 10.1016/j.jad.2022.12.063

[bibr44-00178969241300100] Sharp P Spence JC Bottorff JL , et al. (2020) One small step for man, one giant leap for men’s health: A meta-analysis of behaviour change interventions to increase men’s physical activity. British Journal of Sports Medicine 54(20): 1208–1216. 32024644 10.1136/bjsports-2019-100912

[bibr45-00178969241300100] Silveira EA Mendonça CR Delpino FM , et al. (2022) Sedentary behavior, physical inactivity, abdominal obesity and obesity in adults and older adults: A systematic review and meta-analysis. Clinical Nutrition ESPEN 50: 63–73. 35871953 10.1016/j.clnesp.2022.06.001

[bibr46-00178969241300100] Thompson ER (2007) Development and validation of an internationally reliable short-form of the positive and negative affect schedule (PANAS). Journal of Cross-Cultural Psychology 38(2): 227–242.

[bibr47-00178969241300100] Tse AC Wong TW Lee PH (2015) Effect of low-intensity exercise on physical and cognitive health in older adults: A systematic review. Sports Medicine – Open 1(1): 37. 26512340 10.1186/s40798-015-0034-8 PMC4612316

[bibr48-00178969241300100] Wang JCK Hagger MS (2023) Self-determination theory in physical activity contexts. In: Ryan RM (ed.) The Oxford Handbook of Self-Determination Theory (pp. 740–759). Oxford: Oxford University Press.

[bibr49-00178969241300100] Watson D Tellegen A (1985) Toward a consensual structure of mood. Psychological Bulletin 98(2): 219–235. 3901060 10.1037//0033-2909.98.2.219

[bibr50-00178969241300100] World Health Organization (WHO) (2022) Global Status Report on Physical Activity 2022: Country Profiles. Geneva: World Health Organization.

[bibr51-00178969241300100] Wyke S Hunt K Gray CM , et al. (2015) Football Fans in Training (FFIT): A randomized controlled trial of a gender sensitised weight loss and healthy living programme delivered to men aged 35-65 by Scottish Premier League (SPL) football clubs. Public Health Research 3(2): 1–130.

[bibr52-00178969241300100] Wyke S Bunn C Andersen E , et al. (2019) The effect of a programme to improve men’s sedentary time and physical activity: The European Fans in Training (EuroFIT) randomised controlled trial. PLoS Medicine 16(2): e1002736. 10.1371/journal.pmed.1002736 PMC6363143 30721231

