# Supplementary material for: Individual and socio-psychological characteristics as predictors of physical activity among men living with overweight and obesity taking part in the Aussie Fans in Training weight management behaviour change programme
Source: Health Educ J. 2024 Nov 23;84(3):219–32. doi: 10.1177/00178969241300100 (PMC11952988; doi:10.1177/00178969241300100)
Supplement: sj-docx-1-hej-10.1177_00178969241300100 – Supplemental material for Individual and socio-psychological characteristics as predictors of physical activity among men living with overweight and obesity taking part in the Aussie Fans in Training weight management behaviour change programme [file sj-docx-1-hej-10.1177_00178969241300100.docx]

| **Variable** | 1 | 2 | 3 | 4 | 5 | 6 | 7 | 8 | 9 | 10 | 11 | 12 | 13 | 14 | 15 | 16 | 17 |
| --- | --- | --- | --- | --- | --- | --- | --- | --- | --- | --- | --- | --- | --- | --- | --- | --- | --- |
| Pre-programme |  |  |  |  |  |  |  |  |  |  |  |  |  |  |  |  |  |
| 1. Age (years) | 1 |  |  |  |  |  |  |  |  |  |  |  |  |  |  |  |  |
| 2. Weight (kg) | -0.21* | 1 |  |  |  |  |  |  |  |  |  |  |  |  |  |  |  |
| 3. Need support | -.08 | -.04 | 1 |  |  |  |  |  |  |  |  |  |  |  |  |  |  |
| 4. Need satisfaction | .15 | -.08 | .56** | 1 |  |  |  |  |  |  |  |  |  |  |  |  |  |
| 5. Positive affect | .10 | -.13 | .25** | .47** | 1 |  |  |  |  |  |  |  |  |  |  |  |  |
| 6. Negative affect | -.09 | .20* | -.22* | -.35** | -.28** | 1 |  |  |  |  |  |  |  |  |  |  |  |
| 7. Waking wear time (min/day) | .13 | -.19 | .11 | .03 | .01 | -.03 | 1 |  |  |  |  |  |  |  |  |  |  |
| 8. MVPA (min/day) | -.03 | -.18 | .11 | .16 | .21* | .09 | .02 | 1 |  |  |  |  |  |  |  |  |  |
| 9. Step count | .04 | -.38** | .13 | .15 | .31** | -.02 | .13 | .43** | 1 |  |  |  |  |  |  |  |  |
| Post-programme |  |  |  |  |  |  |  |  |  |  |  |  |  |  |  |  |  |
| 10. Weight (kg) | -.32** | .96** | -.12 | -.10 | -.12 | .27* | -.28** | -.16 | -.32** | 1 |  |  |  |  |  |  |  |
| 11. Need support | -.03 | .14 | .57** | .33** | .22* | -.22* | .12 | .08 | .10 | -.01 | 1 |  |  |  |  |  |  |
| 12. Need satisfaction | -.08 | .23 | .26* | .26* | .20 | -.14 | .07 | -.07 | -.03 | .09 | .64** | 1 |  |  |  |  |  |
| 13. Positive affect | .08 | .05 | .10 | .12 | .51** | -.08 | .11 | .07 | .12 | -.05 | .37** | .49** | 1 |  |  |  |  |
| 14. Negative affect | -.18 | .14 | -.14 | -.13 | -.18 | .67** | -.16 | .21* | .09 | .14 | -.32** | -.21* | -.26* | 1 |  |  |  |
| 15. Waking wear time (min/day) | -.09 | .08 | -.10 | -.11 | -.07 | .01 | .25* | -.16 | -.08 | .09 | .14 | .04 | .11 | -.10 | 1 |  |  |
| 16. MVPA (min/day) | .12 | -.18 | .25* | .08 | .10 | -.06 | .09 | .45** | .28* | -.32** | .36** | .13 | .16 | -.17 | .04 | 1 |  |
| 17. Step count | .20 | -.31* | .25* | .07 | .28* | .03 | .30* | .43** | .65** | -.40** | .20 | .10 | .19 | -.10 | .08 | .58** | 1 |
| * *p* < 0.05 ** *p* < 0.01. | | |  |  |  |  |  |  |  |  |  |  |  |  |  |  |  |
