# Supplementary material for: Individual and socio-psychological characteristics as predictors of physical activity among men living with overweight and obesity taking part in the Aussie Fans in Training weight management behaviour change programme
Source: Health Educ J. 2024 Nov 23;84(3):219–32. doi: 10.1177/00178969241300100 (PMC11952988; doi:10.1177/00178969241300100)
Supplement: sj-docx-2-hej-10.1177_00178969241300100 – Supplemental material for Individual and socio-psychological characteristics as predictors of physical activity among men living with overweight and obesity taking part in the Aussie Fans in Training weight management behaviour change programme [file sj-docx-2-hej-10.1177_00178969241300100.docx]

**Regression analyses**

## Descriptive Statistics

| Mean | | Std. Deviation | N |
| --- | --- | --- | --- |
| steps_post | 14381.03 | 4305.165 | 65 |
| AGwear_pre | 924.20 | 133.327 | 65 |
| steps_pre | 13095.90 | 3942.224 | 65 |
| Age | 44.9769 | 7.46577 | 65 |
| Weight_pre | 110.64 | 18.765 | 65 |
| needsupp_pre | 5.19 | 1.254 | 65 |
| needsat_pre | 3.25 | .606 | 65 |
| posaff_pre | 2.83 | .576 | 65 |
| negaff_pre | 1.70 | .618 | 65 |

**Correlations**

| steps_post | | | AGwear_pre | steps_pre | Age | Weight_pre | needsupp_pre | needsat_pre | posaff_pre |
| --- | --- | --- | --- | --- | --- | --- | --- | --- | --- |
| Pearson Correlation | steps_post | 1.000 | .301 | .652 | .200 | -.312 | .252 | .074 | .283 |
|  | AGwear_pre | .301 | 1.000 | .204 | .070 | -.231 | .089 | -.126 | -.137 |
|  | steps_pre | .652 | .204 | 1.000 | .102 | -.487 | .185 | .078 | .285 |
|  | Age | .200 | .070 | .102 | 1.000 | -.192 | -.158 | .041 | .130 |
|  | Weight_pre | -.312 | -.231 | -.487 | -.192 | 1.000 | -.055 | -.069 | -.019 |
|  | needsupp_pre | .252 | .089 | .185 | -.158 | -.055 | 1.000 | .550 | .297 |
|  | needsat_pre | .074 | -.126 | .078 | .041 | -.069 | .550 | 1.000 | .381 |
|  | posaff_pre | .283 | -.137 | .285 | .130 | -.019 | .297 | .381 | 1.000 |
|  | negaff_pre | .033 | .104 | .004 | .053 | .236 | -.322 | -.383 | -.215 |
| Sig. (1-tailed) | steps_post | . | .007 | <.001 | .055 | .006 | .022 | .278 | .011 |
|  | AGwear_pre | .007 | . | .051 | .291 | .032 | .240 | .160 | .139 |
|  | steps_pre | .000 | .051 | . | .210 | .000 | .070 | .268 | .011 |
|  | Age | .055 | .291 | .210 | . | .063 | .105 | .372 | .151 |

negaff_pre

| Pearson Correlation | steps_post | .033 |
| --- | --- | --- |
|  | AGwear_pre | .104 |
|  | steps_pre | .004 |
|  | Age | .053 |
|  | Weight_pre | .236 |
|  | needsupp_pre | -.322 |
|  | needsat_pre | -.383 |
|  | posaff_pre | -.215 |
|  | negaff_pre | 1.000 |
| Sig. (1-tailed) | steps_post | .398 |
|  | AGwear_pre | .205 |
|  | steps_pre | .487 |
|  | Age | .338 |

N

steps_post AGwear_pre steps_pre

Weight_pre .006 .032 .000

needsupp_pre .022 .240 .070

needsat_pre .278 .160 .268

posaff_pre .011 .139 .011

negaff_pre .398 .205 .487

steps_post 65 65 65

AGwear_pre 65 65 65

steps_pre 65 65 65

Age 65 65 65

Weight_pre 65 65 65

needsupp_pre 65 65 65

needsat_pre 65 65 65

posaff_pre 65 65 65

negaff_pre 65 65 65

Age Weight_pre needsupp_pre needsat_pre posaff_pre

.063 . .333 .294 .441

.105 .333 . .000 .008

.372 .294 .000 . .001

.151 .441 .008 .001 .

.338 .029 .004 .001 .042

65 65 65 65 65

65 65 65 65 65

65 65 65 65 65

65 65 65 65 65

65 65 65 65 65

65 65 65 65 65

65 65 65 65 65

65 65 65 65 65

65 65 65 65 65

negaff_pre

|  | Weight_pre | .029 |
| --- | --- | --- |
|  | needsupp_pre | .004 |
|  | needsat_pre | .001 |
|  | posaff_pre | .042 |
|  | negaff_pre | . |
| N | steps_post | 65 |
|  | AGwear_pre | 65 |
|  | steps_pre | 65 |
|  | Age | 65 |
|  | Weight_pre | 65 |
|  | needsupp_pre | 65 |
|  | needsat_pre | 65 |
|  | posaff_pre | 65 |
|  | negaff_pre | 65 |

## Variables Entered/Removeda

| Variables  Model Entered | | Variables Removed | Method |
| --- | --- | --- | --- |
| 1 | steps_pre, AGwear_preb | . | Enter |
| 2 | Age, Weight_preb | . | Enter |
| 3 | needsupp_preb | . | Enter |
| 4 | needsat_preb | . | Enter |
| 5 | negaff_pre, posaff_preb | . | Enter |

1. Dependent Variable: steps_post
2. All requested variables entered.

## Model Summaryf

| Model R | | R Square | Adjusted R Square | Std. Error of the Estimate | Change Statistics | | | | |
| --- | --- | --- | --- | --- | --- | --- | --- | --- | --- |
|  |  |  |  |  | R Square Change | F Change | df1 | df2 | Sig. F Change |
| 1 | .674a | .454 | .437 | 3231.318 | .454 | 25.803 | 2 | 62 | <.001 |
| 2 | .688b | .473 | .438 | 3227.958 | .019 | 1.065 | 2 | 60 | .351 |
| 3 | .704c | .495 | .452 | 3186.203 | .022 | 2.583 | 1 | 59 | .113 |
| 4 | .705d | .497 | .445 | 3205.946 | .002 | .276 | 1 | 58 | .602 |
| 5 | .713e | .508 | .438 | 3227.233 | .011 | .619 | 2 | 56 | .542 |

1. Predictors: (Constant), steps_pre, AGwear_pre
2. Predictors: (Constant), steps_pre, AGwear_pre, Age, Weight_pre
3. Predictors: (Constant), steps_pre, AGwear_pre, Age, Weight_pre, needsupp_pre
4. Predictors: (Constant), steps_pre, AGwear_pre, Age, Weight_pre, needsupp_pre, needsat_pre
5. Predictors: (Constant), steps_pre, AGwear_pre, Age, Weight_pre, needsupp_pre, needsat_pre, negaff_pre, posaff_pre
6. Dependent Variable: steps_post

## ANOVAa

| Model Sum of Squares | | | df | Mean Square | F | Sig. |
| --- | --- | --- | --- | --- | --- | --- |
| 1 | Regression | 538836357.70 | 2 | 269418178.85 | 25.803 | <.001b |
|  | Residual | 647367926.63 | 62 | 10441418.171 |  |  |
|  | Total | 1186204284.3 | 64 |  |  |  |
| 2 | Regression | 561021445.13 | 4 | 140255361.28 | 13.461 | <.001c |
|  | Residual | 625182839.20 | 60 | 10419713.987 |  |  |
|  | Total | 1186204284.3 | 64 |  |  |  |
| 3 | Regression | 587242658.99 | 5 | 117448531.80 | 11.569 | <.001d |
|  | Residual | 598961625.33 | 59 | 10151891.955 |  |  |
|  | Total | 1186204284.3 | 64 |  |  |  |
| 4 | Regression | 590074894.78 | 6 | 98345815.797 | 9.568 | <.001e |
|  | Residual | 596129389.54 | 58 | 10278092.923 |  |  |
|  | Total | 1186204284.3 | 64 |  |  |  |
| 5 | Regression | 602962598.79 | 8 | 75370324.849 | 7.237 | <.001f |
|  | Residual | 583241685.54 | 56 | 10415030.099 |  |  |
|  | Total | 1186204284.3 | 64 |  |  |  |

1. Dependent Variable: steps_post
2. Predictors: (Constant), steps_pre, AGwear_pre
3. Predictors: (Constant), steps_pre, AGwear_pre, Age, Weight_pre
4. Predictors: (Constant), steps_pre, AGwear_pre, Age, Weight_pre, needsupp_pre
5. Predictors: (Constant), steps_pre, AGwear_pre, Age, Weight_pre, needsupp_pre, needsat_pre
6. Predictors: (Constant), steps_pre, AGwear_pre, Age, Weight_pre, needsupp_pre, needsat_pre, negaff_pre, posaff_pre

| Unstandardized Coefficients | | | | Standardized Coefficients |  |  | 95.0% Confidence Interval for B | | Correlations |
| --- | --- | --- | --- | --- | --- | --- | --- | --- | --- |
| Model |  | B | Std. Error | Beta | t | Sig. | Lower Bound | Upper Bound | Zero-order |
| 1 | (Constant) | 338.595 | 2935.805 |  | .115 | .909 | -5529.996 | 6207.185 |  |
|  | AGwear_pre | 5.663 | 3.095 | .175 | 1.830 | .072 | -.524 | 11.849 | .301 |
|  | steps_pre | .673 | .105 | .616 | 6.427 | <.001 | .463 | .882 | .652 |
| 2 | (Constant) | -5046.833 | 5650.500 |  | -.893 | .375 | -16349.517 | 6255.851 |  |
|  | AGwear_pre | 5.717 | 3.130 | .177 | 1.827 | .073 | -.543 | 11.977 | .301 |
|  | steps_pre | .691 | .118 | .632 | 5.860 | <.001 | .455 | .926 | .652 |
|  | Age | 78.016 | 55.086 | .135 | 1.416 | .162 | -32.173 | 188.204 | .200 |
|  | Weight_pre | 14.386 | 25.225 | .063 | .570 | .571 | -36.073 | 64.844 | -.312 |
| 3 | (Constant) | -7657.973 | 5809.233 |  | -1.318 | .193 | -19282.222 | 3966.276 |  |
|  | AGwear_pre | 5.388 | 3.096 | .167 | 1.740 | .087 | -.806 | 11.583 | .301 |
|  | steps_pre | .657 | .118 | .601 | 5.553 | <.001 | .420 | .893 | .652 |
|  | Age | 93.845 | 55.259 | .163 | 1.698 | .095 | -16.727 | 204.418 | .200 |
|  | Weight_pre | 13.510 | 24.905 | .059 | .542 | .590 | -36.324 | 63.345 | -.312 |
|  | needsupp_pre | 529.270 | 329.324 | .154 | 1.607 | .113 | -129.707 | 1188.246 | .252 |
| 4 | (Constant) | -6600.603 | 6182.557 |  | -1.068 | .290 | -18976.336 | 5775.130 |  |
|  | AGwear_pre | 5.001 | 3.201 | .155 | 1.562 | .124 | -1.407 | 11.409 | .301 |
|  | steps_pre | .654 | .119 | .599 | 5.490 | <.001 | .415 | .892 | .652 |
|  | Age | 98.714 | 56.369 | .171 | 1.751 | .085 | -14.121 | 211.549 | .200 |
|  | Weight_pre | 12.452 | 25.140 | .054 | .495 | .622 | -37.872 | 62.776 | -.312 |
|  | needsupp_pre | 653.387 | 407.072 | .190 | 1.605 | .114 | -161.456 | 1468.229 | .252 |
|  | needsat_pre | -433.204 | 825.246 | -.061 | -.525 | .602 | -2085.114 | 1218.706 | .074 |
| 5 | (Constant) | -7361.608 | 6265.662 |  | -1.175 | .245 | -19913.237 | 5190.022 |  |
|  | AGwear_pre | 5.360 | 3.291 | .166 | 1.629 | .109 | -1.233 | 11.952 | .301 |
|  | steps_pre | .599 | .130 | .549 | 4.616 | <.001 | .339 | .859 | .652 |
|  | Age | 86.528 | 57.795 | .150 | 1.497 | .140 | -29.250 | 202.305 | .200 |
|  | Weight_pre | 3.643 | 27.266 | .016 | .134 | .894 | -50.977 | 58.264 | -.312 |

| Correlations | | | | Collinearity Statistics | |
| --- | --- | --- | --- | --- | --- |
| Model | | Partial | Part | Tolerance | VIF |
| 1 | (Constant) |  |  |  |  |
|  | AGwear_pre | .226 | .172 | .958 | 1.043 |
|  | steps_pre | .632 | .603 | .958 | 1.043 |
| 2 | (Constant) |  |  |  |  |
|  | AGwear_pre | .230 | .171 | .935 | 1.069 |
|  | steps_pre | .603 | .549 | .754 | 1.326 |
|  | Age | .180 | .133 | .963 | 1.039 |
|  | Weight_pre | .073 | .053 | .727 | 1.376 |
| 3 | (Constant) |  |  |  |  |
|  | AGwear_pre | .221 | .161 | .931 | 1.074 |
|  | steps_pre | .586 | .514 | .730 | 1.370 |
|  | Age | .216 | .157 | .932 | 1.073 |
|  | Weight_pre | .070 | .050 | .726 | 1.377 |
|  | needsupp_pre | .205 | .149 | .930 | 1.075 |
| 4 | (Constant) |  |  |  |  |
|  | AGwear_pre | .201 | .145 | .882 | 1.134 |
|  | steps_pre | .585 | .511 | .729 | 1.372 |
|  | Age | .224 | .163 | .907 | 1.103 |
|  | Weight_pre | .065 | .046 | .722 | 1.386 |
|  | needsupp_pre | .206 | .149 | .616 | 1.623 |
|  | needsat_pre | -.069 | -.049 | .642 | 1.558 |
| 5 | (Constant) |  |  |  |  |
|  | AGwear_pre | .213 | .153 | .845 | 1.183 |
|  | steps_pre | .525 | .433 | .622 | 1.608 |
|  | Age | .196 | .140 | .874 | 1.144 |
|  | Weight_pre | .018 | .013 | .622 | 1.609 |

| Unstandardized Coefficients | | | | Standardized Coefficients |  |  | 95.0% Confidence Interval for B | | Correlations |
| --- | --- | --- | --- | --- | --- | --- | --- | --- | --- |
| Model |  | B | Std. Error | Beta | t | Sig. | Lower Bound | Upper Bound | Zero-order |
|  | needsupp_pre | 638.868 | 418.710 | .186 | 1.526 | .133 | -199.908 | 1477.645 | .252 |
|  | needsat_pre | -552.027 | 872.434 | -.078 | -.633 | .529 | -2299.722 | 1195.668 | .074 |
|  | posaff_pre | 868.916 | 829.255 | .116 | 1.048 | .299 | -792.281 | 2530.112 | .283 |
|  | negaff_pre | 394.633 | 766.903 | .057 | .515 | .609 | -1141.658 | 1930.925 | .033 |

## Coefficientsa

| Correlations | | | | Collinearity Statistics | |
| --- | --- | --- | --- | --- | --- |
| Model | | Partial | Part | Tolerance | VIF |
|  | needsupp_pre | .200 | .143 | .590 | 1.694 |
|  | needsat_pre | -.084 | -.059 | .582 | 1.718 |
|  | posaff_pre | .139 | .098 | .712 | 1.404 |
|  | negaff_pre | .069 | .048 | .725 | 1.378 |

1. Dependent Variable: steps_post

## Excluded Variablesa

| Model Beta In | | | t | Sig. | Partial Correlation | Collinearity Statistics | | |
| --- | --- | --- | --- | --- | --- | --- | --- | --- |
|  |  |  |  |  |  | Tolerance | VIF | Minimum Tolerance |
| 1 | Age | .127b | 1.351 | .182 | .170 | .987 | 1.013 | .951 |
|  | Weight_pre | .038b | .348 | .729 | .045 | .745 | 1.342 | .745 |
|  | needsupp_pre | .127b | 1.331 | .188 | .168 | .963 | 1.038 | .930 |
|  | needsat_pre | .050b | .518 | .606 | .066 | .973 | 1.028 | .938 |
|  | posaff_pre | .149b | 1.505 | .137 | .189 | .879 | 1.137 | .859 |
|  | negaff_pre | .012b | .125 | .901 | .016 | .989 | 1.011 | .948 |
| 2 | needsupp_pre | .154c | 1.607 | .113 | .205 | .930 | 1.075 | .726 |
|  | needsat_pre | .047c | .494 | .623 | .064 | .969 | 1.032 | .725 |
|  | posaff_pre | .130c | 1.282 | .205 | .165 | .851 | 1.175 | .668 |
|  | negaff_pre | -.012c | -.117 | .907 | -.015 | .895 | 1.118 | .659 |
| 3 | needsat_pre | -.061d | -.525 | .602 | -.069 | .642 | 1.558 | .616 |
|  | posaff_pre | .089d | .839 | .405 | .109 | .771 | 1.297 | .664 |
|  | negaff_pre | .051d | .485 | .629 | .064 | .780 | 1.282 | .646 |
| 4 | posaff_pre | .108e | .993 | .325 | .130 | .726 | 1.378 | .604 |
|  | negaff_pre | .041e | .373 | .710 | .049 | .740 | 1.352 | .596 |

1. Dependent Variable: steps_post
2. Predictors in the Model: (Constant), steps_pre, AGwear_pre
3. Predictors in the Model: (Constant), steps_pre, AGwear_pre, Age, Weight_pre
4. Predictors in the Model: (Constant), steps_pre, AGwear_pre, Age, Weight_pre, needsupp_pre
5. Predictors in the Model: (Constant), steps_pre, AGwear_pre, Age, Weight_pre, needsupp_pre, needsat_pre

Variance Proportions

Model Dimension Eigenvalue

Condition Index

(Constant)

AGwear_pre

steps_pre

Age

Weight_pre

needsupp_pre

needsat_pre

1 1 2.937

2 .053

3 .010

2 1 4.867

2 .083

3 .027

4 .020

5 .004

3 1 5.820

2 .083

3 .050

4 .024

5 .019

6 .004

4 1 6.794

2 .083

3 .056

4 .027

5 .022

6 .014

7 .003

5 1 8.649

2 .142

3 .082

4 .040

5 .032

1.000

7.425

17.072

1.000

7.674

13.530

15.676

35.402

1.000

8.392

10.804

15.474

17.317

39.763

1.000

9.022

11.016

15.775

17.553

22.114

45.143

1.000

7.809

10.272

14.699

16.480

.00

.05

.94

.00

.00

.00

.00

1.00

.00

.00

.00

.00

.00

1.00

.00

.00

.00

.00

.00

.00

.99

.00

.00

.00

.00

.00

.00

.06

.93

.00

.00

.00

.69

.30

.00

.00

.01

.02

.71

.27

.00

.00

.02

.04

.42

.21

.32

.00

.00

.00

.00

.20

.01

.99

.00

.00

.50

.18

.14

.18

.00

.49

.02

.31

.06

.13

.00

.50

.01

.09

.26

.00

.13

.00

.01

.43

.01

.01

.00

.00

.60

.17

.23

.00

.00

.10

.39

.24

.26

.00

.00

.08

.37

.01

.38

.16

.00

.00

.00

.22

.05

.00

.07

.22

.10

.61

.00

.07

.00

.32

.04

.56

.00

.06

.01

.18

.18

.02

.54

.00

.00

.04

.00

.01

.00

.00

.76

.14

.04

.06

.00

.00

.33

.02

.11

.54

.00

.00

.03

.01

.36

.02

.00

.00

.05

.17

.09

.56

.12

.00

.01

.01

.00

.00

Variance Proportions

| Model Dimension | | posaff_pre | negaff_pre |
| --- | --- | --- | --- |
| 1 | 1 |  |  |
|  | 2 |  |  |
|  | 3 |  |  |
| 2 | 1 |  |  |
|  | 2 |  |  |
|  | 3 |  |  |
|  | 4 |  |  |
|  | 5 |  |  |
| 3 | 1 |  |  |
|  | 2 |  |  |
|  | 3 |  |  |
|  | 4 |  |  |
|  | 5 |  |  |
|  | 6 |  |  |
| 4 | 1 |  |  |
|  | 2 |  |  |
|  | 3 |  |  |
|  | 4 |  |  |
|  | 5 |  |  |
|  | 6 |  |  |
|  | 7 |  |  |
| 5 | 1 | .00 | .00 |
|  | 2 | .01 | .40 |
|  | 3 | .00 | .01 |
|  | 4 | .05 | .17 |
|  | 5 | .41 | .08 |

Variance Proportions

Model Dimension Eigenvalue Condition Index (Constant) AGwear_pre steps_pre

6 .022 19.799 .00 .07 .10

7 .016 22.964 .00 .15 .30

8 .014 25.231 .00 .27 .05

9 .003 50.972 .99 .31 .10

Age Weight_pre needsupp_pre needsat_pre

.20 .26 .04 .18

.00 .15 .15 .29

.38 .09 .40 .39

.15 .45 .00 .10

## Collinearity Diagnosticsa

Variance Proportions

| Model Dimension | | posaff_pre | negaff_pre |
| --- | --- | --- | --- |
|  | 6 | .01 | .28 |
|  | 7 | .47 | .03 |
|  | 8 | .06 | .03 |
|  | 9 | .00 | .00 |

1. Dependent Variable: steps_post

## Residuals Statisticsa

| Minimum | | Maximum | Mean | Std. Deviation | N |
| --- | --- | --- | --- | --- | --- |
| Predicted Value | 7196.58 | 23332.63 | 14381.03 | 3069.412 | 65 |
| Std. Predicted Value | -2.341 | 2.916 | .000 | 1.000 | 65 |
| Standard Error of Predicted Value | 612.758 | 1922.312 | 1168.019 | 281.120 | 65 |
| Adjusted Predicted Value | 6964.03 | 23147.39 | 14372.94 | 3084.017 | 65 |
| Residual | -6564.571 | 8398.196 | .000 | 3018.800 | 65 |
| Std. Residual | -2.034 | 2.602 | .000 | .935 | 65 |
| Stud. Residual | -2.163 | 2.971 | .001 | 1.011 | 65 |
| Deleted Residual | -7426.221 | 10946.919 | 8.086 | 3534.244 | 65 |
| Stud. Deleted Residual | -2.240 | 3.208 | .003 | 1.033 | 65 |
| Mahal. Distance | 1.323 | 21.723 | 7.877 | 4.129 | 65 |
| Cook's Distance | .000 | .298 | .019 | .040 | 65 |
| Centered Leverage Value | .021 | .339 | .123 | .065 | 65 |

1. Dependent Variable: steps_post

**Charts**

# Normal P-P Plot of Regression Standardized Residual Dependent Variable: steps_post

1.0


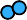

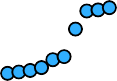

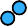

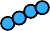

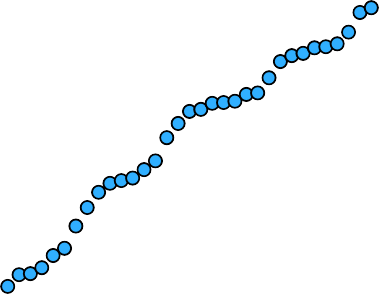

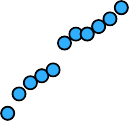

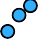


0.8

**Expected Cum Prob**

0.6

0.4

0.2

0.0

0.0

0.2

0.4

0.6

0.8

1.0

# Observed Cum Prob

**Scatterplot Dependent Variable: steps_post**

3


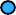

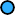

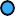

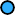

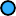

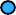

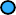

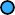

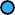

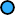

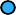

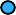

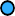

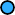

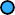

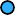

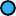

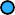

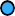

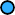

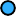

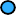

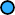

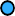

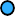

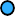

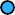

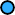

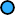

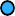

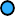

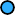

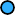

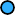

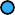

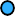

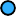

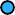

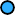

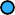

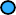

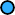

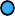

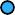

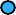

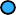

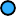

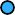

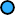

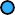

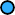


2

**Regression Standardized Residual**

1

0

-1

-2

-3

-3 -2 -1 0 1 2 3

# Regression Standardized Predicted Value

**Regression**

## Descriptive Statistics

| Mean | | Std. Deviation | N |
| --- | --- | --- | --- |
| MVPA_post | 45.32 | 21.594 | 65 |
| AGwear_pre | 924.20 | 133.327 | 65 |
| MVPA_pre | 36.48 | 19.655 | 65 |
| Age | 44.9769 | 7.46577 | 65 |
| Weight_pre | 110.64 | 18.765 | 65 |
| needsupp_pre | 5.19 | 1.254 | 65 |
| needsat_pre | 3.25 | .606 | 65 |
| posaff_pre | 2.83 | .576 | 65 |
| negaff_pre | 1.70 | .618 | 65 |

**Correlations**

| MVPA_post | | | AGwear_pre | MVPA_pre | Age | Weight_pre | needsupp_pre | needsat_pre | posaff_pre |
| --- | --- | --- | --- | --- | --- | --- | --- | --- | --- |
| Pearson Correlation | MVPA_post | 1.000 | .091 | .446 | .120 | -.176 | .254 | .082 | .102 |
|  | AGwear_pre | .091 | 1.000 | .094 | .070 | -.231 | .089 | -.126 | -.137 |
|  | MVPA_pre | .446 | .094 | 1.000 | .142 | -.318 | .232 | .198 | .209 |
|  | Age | .120 | .070 | .142 | 1.000 | -.192 | -.158 | .041 | .130 |
|  | Weight_pre | -.176 | -.231 | -.318 | -.192 | 1.000 | -.055 | -.069 | -.019 |
|  | needsupp_pre | .254 | .089 | .232 | -.158 | -.055 | 1.000 | .550 | .297 |
|  | needsat_pre | .082 | -.126 | .198 | .041 | -.069 | .550 | 1.000 | .381 |
|  | posaff_pre | .102 | -.137 | .209 | .130 | -.019 | .297 | .381 | 1.000 |
|  | negaff_pre | -.062 | .104 | .017 | .053 | .236 | -.322 | -.383 | -.215 |
| Sig. (1-tailed) | MVPA_post | . | .235 | <.001 | .170 | .081 | .021 | .258 | .210 |
|  | AGwear_pre | .235 | . | .228 | .291 | .032 | .240 | .160 | .139 |
|  | MVPA_pre | .000 | .228 | . | .130 | .005 | .032 | .057 | .048 |
|  | Age | .170 | .291 | .130 | . | .063 | .105 | .372 | .151 |
|  | Weight_pre | .081 | .032 | .005 | .063 | . | .333 | .294 | .441 |
|  | needsupp_pre | .021 | .240 | .032 | .105 | .333 | . | .000 | .008 |

negaff_pre

| Pearson Correlation | MVPA_post | -.062 |
| --- | --- | --- |
|  | AGwear_pre | .104 |
|  | MVPA_pre | .017 |
|  | Age | .053 |
|  | Weight_pre | .236 |
|  | needsupp_pre | -.322 |
|  | needsat_pre | -.383 |
|  | posaff_pre | -.215 |
|  | negaff_pre | 1.000 |
| Sig. (1-tailed) | MVPA_post | .311 |
|  | AGwear_pre | .205 |
|  | MVPA_pre | .447 |
|  | Age | .338 |
|  | Weight_pre | .029 |
|  | needsupp_pre | .004 |

N

MVPA_post AGwear_pre MVPA_pre

needsat_pre .258 .160 .057

posaff_pre .210 .139 .048

negaff_pre .311 .205 .447

MVPA_post 65 65 65

AGwear_pre 65 65 65

MVPA_pre 65 65 65

Age 65 65 65

Weight_pre 65 65 65

needsupp_pre 65 65 65

needsat_pre 65 65 65

posaff_pre 65 65 65

negaff_pre 65 65 65

Age Weight_pre needsupp_pre needsat_pre posaff_pre

.372 .294 .000 . .001

.151 .441 .008 .001 .

.338 .029 .004 .001 .042

65 65 65 65 65

65 65 65 65 65

65 65 65 65 65

65 65 65 65 65

65 65 65 65 65

65 65 65 65 65

65 65 65 65 65

65 65 65 65 65

65 65 65 65 65

## Correlations

negaff_pre

|  | needsat_pre | .001 |
| --- | --- | --- |
|  | posaff_pre | .042 |
|  | negaff_pre | . |
| N | MVPA_post | 65 |
|  | AGwear_pre | 65 |
|  | MVPA_pre | 65 |
|  | Age | 65 |
|  | Weight_pre | 65 |
|  | needsupp_pre | 65 |
|  | needsat_pre | 65 |
|  | posaff_pre | 65 |
|  | negaff_pre | 65 |

## Variables Entered/Removeda

| Variables  Model Entered | | Variables Removed | Method |
| --- | --- | --- | --- |
| 1 | MVPA_pre, AGwear_preb | . | Enter |
| 2 | Age, Weight_preb | . | Enter |
| 3 | needsupp_preb | . | Enter |
| 4 | needsat_preb | . | Enter |
| 5 | posaff_pre, negaff_preb | . | Enter |

1. Dependent Variable: MVPA_post
2. All requested variables entered.

## Model Summaryf

| Model R | | R Square | Adjusted R Square | Std. Error of the Estimate | Change Statistics | | | | |
| --- | --- | --- | --- | --- | --- | --- | --- | --- | --- |
|  |  |  |  |  | R Square Change | F Change | df1 | df2 | Sig. F Change |
| 1 | .449a | .202 | .176 | 19.604 | .202 | 7.827 | 2 | 62 | <.001 |
| 2 | .453b | .205 | .152 | 19.886 | .003 | .127 | 2 | 60 | .881 |
| 3 | .482c | .233 | .168 | 19.702 | .028 | 2.129 | 1 | 59 | .150 |
| 4 | .496d | .246 | .168 | 19.700 | .013 | 1.012 | 1 | 58 | .319 |
| 5 | .498e | .248 | .141 | 20.012 | .003 | .100 | 2 | 56 | .905 |

1. Predictors: (Constant), MVPA_pre, AGwear_pre
2. Predictors: (Constant), MVPA_pre, AGwear_pre, Age, Weight_pre
3. Predictors: (Constant), MVPA_pre, AGwear_pre, Age, Weight_pre, needsupp_pre
4. Predictors: (Constant), MVPA_pre, AGwear_pre, Age, Weight_pre, needsupp_pre, needsat_pre
5. Predictors: (Constant), MVPA_pre, AGwear_pre, Age, Weight_pre, needsupp_pre, needsat_pre, posaff_pre, negaff_pre
6. Dependent Variable: MVPA_post

## ANOVAa

| Model Sum of Squares | | | df | Mean Square | F | Sig. |
| --- | --- | --- | --- | --- | --- | --- |
| 1 | Regression | 6016.312 | 2 | 3008.156 | 7.827 | <.001b |
|  | Residual | 23827.373 | 62 | 384.312 |  |  |
|  | Total | 29843.685 | 64 |  |  |  |
| 2 | Regression | 6116.367 | 4 | 1529.092 | 3.867 | .007c |
|  | Residual | 23727.319 | 60 | 395.455 |  |  |
|  | Total | 29843.685 | 64 |  |  |  |
| 3 | Regression | 6942.773 | 5 | 1388.555 | 3.577 | .007d |
|  | Residual | 22900.912 | 59 | 388.151 |  |  |
|  | Total | 29843.685 | 64 |  |  |  |
| 4 | Regression | 7335.562 | 6 | 1222.594 | 3.150 | .010e |
|  | Residual | 22508.123 | 58 | 388.071 |  |  |
|  | Total | 29843.685 | 64 |  |  |  |
| 5 | Regression | 7415.903 | 8 | 926.988 | 2.315 | .032f |
|  | Residual | 22427.783 | 56 | 400.496 |  |  |
|  | Total | 29843.685 | 64 |  |  |  |

1. Dependent Variable: MVPA_post
2. Predictors: (Constant), MVPA_pre, AGwear_pre
3. Predictors: (Constant), MVPA_pre, AGwear_pre, Age, Weight_pre
4. Predictors: (Constant), MVPA_pre, AGwear_pre, Age, Weight_pre, needsupp_pre
5. Predictors: (Constant), MVPA_pre, AGwear_pre, Age, Weight_pre, needsupp_pre, needsat_pre
6. Predictors: (Constant), MVPA_pre, AGwear_pre, Age, Weight_pre, needsupp_pre, needsat_pre, posaff_pre, negaff_pre

| Unstandardized Coefficients | | | | Standardized Coefficients |  |  | 95.0% Confidence Interval for B | | Correlations |
| --- | --- | --- | --- | --- | --- | --- | --- | --- | --- |
| Model |  | B | Std. Error | Beta | t | Sig. | Lower Bound | Upper Bound | Zero-order |
| 1 | (Constant) | 20.193 | 17.414 |  | 1.160 | .251 | -14.616 | 55.003 |  |
|  | AGwear_pre | .008 | .018 | .050 | .435 | .665 | -.029 | .045 | .091 |
|  | MVPA_pre | .485 | .125 | .442 | 3.874 | <.001 | .235 | .735 | .446 |
| 2 | (Constant) | 17.387 | 32.234 |  | .539 | .592 | -47.090 | 81.864 |  |
|  | AGwear_pre | .007 | .019 | .043 | .360 | .720 | -.031 | .045 | .091 |
|  | MVPA_pre | .471 | .134 | .429 | 3.516 | <.001 | .203 | .739 | .446 |
|  | Age | .153 | .341 | .053 | .450 | .655 | -.528 | .834 | .120 |
|  | Weight_pre | -.023 | .145 | -.020 | -.157 | .876 | -.312 | .266 | -.176 |
| 3 | (Constant) | 1.290 | 33.786 |  | .038 | .970 | -66.316 | 68.897 |  |
|  | AGwear_pre | .005 | .019 | .029 | .242 | .809 | -.034 | .043 | .091 |
|  | MVPA_pre | .422 | .137 | .384 | 3.082 | .003 | .148 | .696 | .446 |
|  | Age | .253 | .344 | .088 | .736 | .465 | -.436 | .943 | .120 |
|  | Weight_pre | -.024 | .143 | -.021 | -.168 | .867 | -.311 | .263 | -.176 |
|  | needsupp_pre | 3.015 | 2.067 | .175 | 1.459 | .150 | -1.120 | 7.151 | .254 |
| 4 | (Constant) | 12.870 | 35.690 |  | .361 | .720 | -58.571 | 84.312 |  |
|  | AGwear_pre | -2.730e-5 | .020 | .000 | -.001 | .999 | -.039 | .039 | .091 |
|  | MVPA_pre | .430 | .137 | .391 | 3.137 | .003 | .156 | .704 | .446 |
|  | Age | .307 | .349 | .106 | .882 | .381 | -.390 | 1.005 | .120 |
|  | Weight_pre | -.031 | .143 | -.027 | -.216 | .830 | -.318 | .256 | -.176 |
|  | needsupp_pre | 4.431 | 2.500 | .257 | 1.772 | .082 | -.573 | 9.436 | .254 |
|  | needsat_pre | -5.105 | 5.075 | -.143 | -1.006 | .319 | -15.263 | 5.053 | .082 |
| 5 | (Constant) | 16.180 | 37.284 |  | .434 | .666 | -58.508 | 90.868 |  |
|  | AGwear_pre | .001 | .020 | .004 | .034 | .973 | -.040 | .041 | .091 |
|  | MVPA_pre | .446 | .144 | .406 | 3.100 | .003 | .158 | .734 | .446 |
|  | Age | .327 | .359 | .113 | .911 | .366 | -.393 | 1.047 | .120 |
|  | Weight_pre | -.009 | .154 | -.008 | -.060 | .952 | -.318 | .299 | -.176 |

| Correlations | | | | Collinearity Statistics | |
| --- | --- | --- | --- | --- | --- |
| Model | | Partial | Part | Tolerance | VIF |
| 1 | (Constant) |  |  |  |  |
|  | AGwear_pre | .055 | .049 | .991 | 1.009 |
|  | MVPA_pre | .441 | .440 | .991 | 1.009 |
| 2 | (Constant) |  |  |  |  |
|  | AGwear_pre | .046 | .041 | .946 | 1.057 |
|  | MVPA_pre | .413 | .405 | .892 | 1.121 |
|  | Age | .058 | .052 | .955 | 1.047 |
|  | Weight_pre | -.020 | -.018 | .840 | 1.191 |
| 3 | (Constant) |  |  |  |  |
|  | AGwear_pre | .032 | .028 | .939 | 1.065 |
|  | MVPA_pre | .372 | .352 | .838 | 1.193 |
|  | Age | .095 | .084 | .917 | 1.090 |
|  | Weight_pre | -.022 | -.019 | .840 | 1.191 |
|  | needsupp_pre | .187 | .166 | .903 | 1.107 |
| 4 | (Constant) |  |  |  |  |
|  | AGwear_pre | .000 | .000 | .887 | 1.127 |
|  | MVPA_pre | .381 | .358 | .835 | 1.197 |
|  | Age | .115 | .101 | .896 | 1.117 |
|  | Weight_pre | -.028 | -.025 | .838 | 1.194 |
|  | needsupp_pre | .227 | .202 | .617 | 1.621 |
|  | needsat_pre | -.131 | -.115 | .641 | 1.560 |
| 5 | (Constant) |  |  |  |  |
|  | AGwear_pre | .005 | .004 | .854 | 1.171 |
|  | MVPA_pre | .383 | .359 | .783 | 1.277 |
|  | Age | .121 | .105 | .869 | 1.150 |
|  | Weight_pre | -.008 | -.007 | .751 | 1.332 |

| Unstandardized Coefficients | | | | Standardized Coefficients |  |  | 95.0% Confidence Interval for B | | Correlations |
| --- | --- | --- | --- | --- | --- | --- | --- | --- | --- |
| Model |  | B | Std. Error | Beta | t | Sig. | Lower Bound | Upper Bound | Zero-order |
|  | needsupp_pre | 4.306 | 2.606 | .250 | 1.652 | .104 | -.915 | 9.527 | .254 |
|  | needsat_pre | -5.443 | 5.413 | -.153 | -1.006 | .319 | -16.286 | 5.400 | .082 |
|  | posaff_pre | -.951 | 4.893 | -.025 | -.194 | .847 | -10.753 | 8.850 | .102 |
|  | negaff_pre | -1.995 | 4.745 | -.057 | -.420 | .676 | -11.501 | 7.511 | -.062 |

## Coefficientsa

| Correlations | | | | Collinearity Statistics | |
| --- | --- | --- | --- | --- | --- |
| Model | | Partial | Part | Tolerance | VIF |
|  | needsupp_pre | .216 | .191 | .586 | 1.707 |
|  | needsat_pre | -.133 | -.116 | .581 | 1.720 |
|  | posaff_pre | -.026 | -.023 | .786 | 1.271 |
|  | negaff_pre | -.056 | -.049 | .729 | 1.372 |

1. Dependent Variable: MVPA_post

## Excluded Variablesa

| Model Beta In | | | t | Sig. | Partial Correlation | Collinearity Statistics | | |
| --- | --- | --- | --- | --- | --- | --- | --- | --- |
|  |  |  |  |  |  | Tolerance | VIF | Minimum Tolerance |
| 1 | Age | .056b | .482 | .632 | .062 | .977 | 1.024 | .973 |
|  | Weight_pre | -.028b | -.227 | .821 | -.029 | .858 | 1.165 | .858 |
|  | needsupp_pre | .156b | 1.345 | .183 | .170 | .942 | 1.062 | .941 |
|  | needsat_pre | .001b | .007 | .995 | .001 | .940 | 1.064 | .940 |
|  | posaff_pre | .018b | .150 | .882 | .019 | .932 | 1.073 | .932 |
|  | negaff_pre | -.076b | -.661 | .511 | -.084 | .989 | 1.011 | .981 |
| 2 | needsupp_pre | .175c | 1.459 | .150 | .187 | .903 | 1.107 | .838 |
|  | needsat_pre | -.001c | -.010 | .992 | -.001 | .938 | 1.066 | .839 |
|  | posaff_pre | .012c | .099 | .921 | .013 | .918 | 1.089 | .839 |
|  | negaff_pre | -.080c | -.657 | .514 | -.085 | .901 | 1.109 | .767 |
| 3 | needsat_pre | -.143d | -1.006 | .319 | -.131 | .641 | 1.560 | .617 |
|  | posaff_pre | -.046d | -.364 | .717 | -.048 | .831 | 1.203 | .817 |
|  | negaff_pre | -.019d | -.147 | .884 | -.019 | .784 | 1.276 | .756 |
| 4 | posaff_pre | -.020e | -.156 | .877 | -.021 | .794 | 1.260 | .603 |
|  | negaff_pre | -.054e | -.407 | .686 | -.054 | .736 | 1.359 | .596 |

1. Dependent Variable: MVPA_post
2. Predictors in the Model: (Constant), MVPA_pre, AGwear_pre
3. Predictors in the Model: (Constant), MVPA_pre, AGwear_pre, Age, Weight_pre
4. Predictors in the Model: (Constant), MVPA_pre, AGwear_pre, Age, Weight_pre, needsupp_pre
5. Predictors in the Model: (Constant), MVPA_pre, AGwear_pre, Age, Weight_pre, needsupp_pre, needsat_pre

Variance Proportions

Model Dimension Eigenvalue

Condition Index

(Constant)

AGwear_pre

MVPA_pre

Age

Weight_pre

needsupp_pre

needsat_pre

1 1 2.835

2 .155

3 .010

2 1 4.751

2 .193

3 .030

4 .021

5 .004

3 1 5.703

2 .194

3 .049

4 .029

5 .020

6 .004

4 1 6.677

2 .196

3 .055

4 .030

5 .025

6 .014

7 .004

5 1 8.528

2 .204

3 .134

4 .040

5 .032

1.000

4.279

16.775

1.000

4.957

12.524

14.870

32.795

1.000

5.422

10.751

13.930

16.999

37.313

1.000

5.842

11.045

15.022

16.310

21.909

42.329

1.000

6.460

7.981

14.516

16.221

.00

.02

.98

.00

.00

.00

.00

1.00

.00

.00

.00

.00

.00

1.00

.00

.00

.00

.00

.00

.00

.99

.00

.00

.00

.00

.00

.00

.02

.98

.00

.00

.07

.49

.44

.00

.00

.01

.10

.54

.36

.00

.00

.02

.05

.33

.18

.42

.00

.00

.00

.00

.21

.02

.97

.00

.01

.82

.12

.00

.05

.00

.79

.02

.16

.01

.02

.00

.80

.03

.16

.00

.00

.01

.00

.68

.09

.00

.01

.00

.00

.26

.49

.25

.00

.00

.11

.13

.47

.29

.00

.00

.09

.17

.14

.41

.18

.00

.00

.00

.23

.03

.00

.01

.43

.03

.53

.00

.01

.00

.50

.00

.49

.00

.01

.01

.50

.00

.02

.45

.00

.01

.00

.01

.06

.00

.00

.74

.05

.13

.08

.00

.00

.34

.01

.08

.57

.00

.00

.00

.03

.33

.04

.00

.00

.05

.01

.26

.55

.12

.00

.00

.02

.00

.00

Variance Proportions

| Model Dimension | | posaff_pre | negaff_pre |
| --- | --- | --- | --- |
| 1 | 1 |  |  |
|  | 2 |  |  |
|  | 3 |  |  |
| 2 | 1 |  |  |
|  | 2 |  |  |
|  | 3 |  |  |
|  | 4 |  |  |
|  | 5 |  |  |
| 3 | 1 |  |  |
|  | 2 |  |  |
|  | 3 |  |  |
|  | 4 |  |  |
|  | 5 |  |  |
|  | 6 |  |  |
| 4 | 1 |  |  |
|  | 2 |  |  |
|  | 3 |  |  |
|  | 4 |  |  |
|  | 5 |  |  |
|  | 6 |  |  |
|  | 7 |  |  |
| 5 | 1 | .00 | .00 |
|  | 2 | .00 | .03 |
|  | 3 | .01 | .39 |
|  | 4 | .05 | .14 |
|  | 5 | .37 | .03 |

Variance Proportions

Model Dimension Eigenvalue Condition Index (Constant) AGwear_pre MVPA_pre

6 .024 18.992 .00 .00 .20

7 .020 20.783 .00 .22 .01

8 .014 24.818 .00 .16 .00

9 .004 48.516 .99 .41 .00

Age Weight_pre needsupp_pre needsat_pre

.01 .53 .08 .00

.17 .01 .00 .37

.41 .03 .52 .51

.15 .35 .00 .09

## Collinearity Diagnosticsa

Variance Proportions

| Model Dimension | | posaff_pre | negaff_pre |
| --- | --- | --- | --- |
|  | 6 | .24 | .32 |
|  | 7 | .30 | .08 |
|  | 8 | .00 | .01 |
|  | 9 | .03 | .01 |

1. Dependent Variable: MVPA_post

## Residuals Statisticsa

| Minimum | | Maximum | Mean | Std. Deviation | N |
| --- | --- | --- | --- | --- | --- |
| Predicted Value | 22.56 | 78.07 | 45.32 | 10.764 | 65 |
| Std. Predicted Value | -2.115 | 3.042 | .000 | 1.000 | 65 |
| Standard Error of Predicted Value | 3.856 | 11.850 | 7.220 | 1.840 | 65 |
| Adjusted Predicted Value | 21.25 | 82.27 | 45.57 | 11.097 | 65 |
| Residual | -45.537 | 40.951 | .000 | 18.720 | 65 |
| Std. Residual | -2.275 | 2.046 | .000 | .935 | 65 |
| Stud. Residual | -2.516 | 2.325 | -.006 | 1.009 | 65 |
| Deleted Residual | -55.697 | 52.845 | -.243 | 21.866 | 65 |
| Stud. Deleted Residual | -2.648 | 2.424 | -.004 | 1.027 | 65 |
| Mahal. Distance | 1.391 | 21.456 | 7.877 | 4.516 | 65 |
| Cook's Distance | .000 | .174 | .019 | .036 | 65 |
| Centered Leverage Value | .022 | .335 | .123 | .071 | 65 |

1. Dependent Variable: MVPA_post

**Charts**

# Normal P-P Plot of Regression Standardized Residual Dependent Variable: MVPA_post

1.0


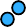

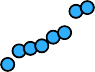

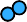

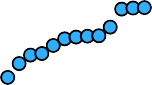

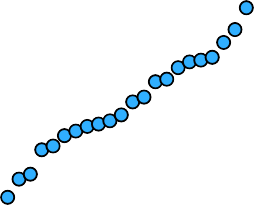

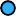

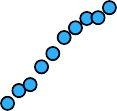

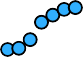


0.8

**Expected Cum Prob**

0.6

0.4

0.2

0.0

0.0

0.2

0.4

0.6

0.8

1.0

# Observed Cum Prob

**Scatterplot Dependent Variable: MVPA_post**

3


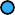

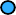

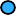

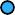

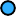

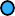

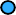

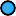

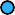

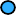

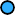

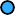

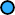

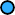

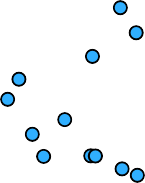

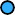

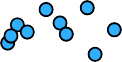

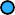

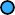

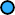

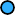

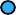

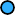

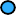

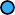

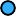

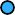

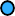

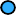

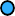

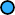

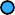

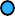

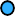


2

**Regression Standardized Residual**

1

0

-1

-2

-3

-2 0 2 4

# Regression Standardized Predicted Value

**Regression**

## Descriptive Statistics

| Mean | | Std. Deviation | N |
| --- | --- | --- | --- |
| steps_post | 14410.37 | 4332.641 | 64 |
| AGwear_pre | 924.55 | 134.352 | 64 |
| AGwear_post | 938.53 | 161.837 | 64 |
| steps_pre | 12985.66 | 3871.075 | 64 |
| Weight_pre | 110.84 | 18.845 | 64 |
| needsupp_pre | 5.16 | 1.243 | 64 |
| needsat_pre | 3.24 | .609 | 64 |
| posaff_pre | 2.83 | .581 | 64 |
| negaff_pre | 1.71 | .619 | 64 |
| Age | 45.0547 | 7.49821 | 64 |
| Weight_post | 107.59 | 18.151 | 64 |
| needsupp_post | 5.56 | 1.125 | 64 |
| needsat_post | 3.89 | .686 | 64 |
| posaff_post | 3.48 | .637 | 64 |
| negaff_post | 1.48 | .485 | 64 |

Pearson Correlation steps_post

AGwear_pre AGwear_post steps_pre Weight_pre needsupp_pre needsat_pre posaff_pre negaff_pre Age Weight_post needsupp_post needsat_post posaff_post negaff_post

Sig. (1-tailed) steps_post AGwear_pre AGwear_post steps_pre Weight_pre needsupp_pre needsat_pre posaff_pre negaff_pre Age Weight_post

needsupp_post

steps_post

1.000

.300

.075

.683

-.318

.266

.080

.283

.027

.196

-.397

.200

.100

.192

-.100

.

.008

.277

.000

.005

.017

.266

.012

.416

.060

.001

.056

AGwear_pre

.300

1.000

.260

.214

-.233

.094

-.124

-.137

.102

.068

-.239

.113

.057

.124

-.099

.008

.

.019

.044

.032

.229

.164

.141

.210

.296

.029

.188

AGwear_post

.075

.260

1.000

-.034

.054

-.044

-.040

.091

-.053

.086

.065

.183

.110

.242

-.153

.277

.019

.

.394

.335

.364

.376

.238

.339

.250

.306

.074

steps_pre

.683

.214

-.034

1.000

-.482

.150

.061

.294

.028

.124

-.472

-.031

-.140

.129

.019

<.001

.044

.394

.

.000

.118

.317

.009

.412

.164

.000

.403

Weight_pre

-.318

-.233

.054

-.482

1.000

-.040

-.062

-.019

.229

-.200

.961

.229

.305

.092

.108

.005

.032

.335

.000

.

.377

.314

.440

.034

.056

.000

.035

needsupp_pre

.266

.094

-.044

.150

-.040

1.000

.545

.303

-.310

-.145

-.077

.560

.214

.109

-.266

.017

.229

.364

.118

.377

.

.000

.007

.006

.126

.273

.000

needsat_pre

.080

-.124

-.040

.061

-.062

.545

1.000

.383

-.378

.049

-.051

.252

.184

.081

-.299

.266

.164

.376

.317

.314

.000

.

.001

.001

.350

.346

.022

Pearson Correlation steps_post

AGwear_pre AGwear_post steps_pre Weight_pre needsupp_pre needsat_pre posaff_pre negaff_pre Age Weight_post needsupp_post needsat_post posaff_post negaff_post

Sig. (1-tailed) steps_post AGwear_pre AGwear_post steps_pre Weight_pre needsupp_pre needsat_pre posaff_pre negaff_pre Age Weight_post

needsupp_post

posaff_pre

.283

-.137

.091

.294

-.019

.303

.383

1.000

-.217

.130

-.062

.242

.178

.437

-.122

.012

.141

.238

.009

.440

.007

.001

.

.042

.153

.313

.027

negaff_pre

.027

.102

-.053

.028

.229

-.310

-.378

-.217

1.000

.044

.286

-.261

-.141

-.004

.660

.416

.210

.339

.412

.034

.006

.001

.042

.

.364

.011

.019

Age

.196

.068

.086

.124

-.200

-.145

.049

.130

.044

1.000

-.236

-.021

-.103

.122

-.204

.060

.296

.250

.164

.056

.126

.350

.153

.364

.

.030

.436

Weight_post

-.397

-.239

.065

-.472

.961

-.077

-.051

-.062

.286

-.236

1.000

.071

.162

-.025

.212

<.001

.029

.306

.000

.000

.273

.346

.313

.011

.030

.

.290

needsupp_post

.200

.113

.183

-.031

.229

.560

.252

.242

-.261

-.021

.071

1.000

.624

.457

-.511

.056

.188

.074

.403

.035

.000

.022

.027

.019

.436

.290

.

needsat_post

.100

.057

.110

-.140

.305

.214

.184

.178

-.141

-.103

.162

.624

1.000

.546

-.395

.217

.326

.194

.135

.007

.045

.073

.079

.133

.208

.100

.000

posaff_post

.192

.124

.242

.129

.092

.109

.081

.437

-.004

.122

-.025

.457

.546

1.000

-.236

.064

.164

.027

.155

.236

.195

.262

.000

.487

.168

.422

.000

negaff_post

| Pearson Correlation | steps_post | -.100 |
| --- | --- | --- |
|  | AGwear_pre | -.099 |
|  | AGwear_post | -.153 |
|  | steps_pre | .019 |
|  | Weight_pre | .108 |
|  | needsupp_pre | -.266 |
|  | needsat_pre | -.299 |
|  | posaff_pre | -.122 |
|  | negaff_pre | .660 |
|  | Age | -.204 |
|  | Weight_post | .212 |
|  | needsupp_post | -.511 |
|  | needsat_post | -.395 |
|  | posaff_post | -.236 |
|  | negaff_post | 1.000 |
| Sig. (1-tailed) | steps_post | .215 |
|  | AGwear_pre | .219 |
|  | AGwear_post | .114 |
|  | steps_pre | .440 |
|  | Weight_pre | .198 |
|  | needsupp_pre | .017 |
|  | needsat_pre | .008 |
|  | posaff_pre | .168 |
|  | negaff_pre | .000 |
|  | Age | .053 |
|  | Weight_post | .047 |
|  | needsupp_post | .000 |

N

steps_post AGwear_pre AGwear_post steps_pre Weight_pre needsupp_pre needsat_pre needsat_post .217 .326 .194 .135 .007 .045 .073

posaff_post .064 .164 .027 .155 .236 .195 .262

negaff_post .215 .219 .114 .440 .198 .017 .008

steps_post 64 64 64 64 64 64 64

AGwear_pre 64 64 64 64 64 64 64

AGwear_post 64 64 64 64 64 64 64

steps_pre 64 64 64 64 64 64 64

Weight_pre 64 64 64 64 64 64 64

needsupp_pre 64 64 64 64 64 64 64

needsat_pre 64 64 64 64 64 64 64

posaff_pre 64 64 64 64 64 64 64

negaff_pre 64 64 64 64 64 64 64

Age 64 64 64 64 64 64 64

Weight_post 64 64 64 64 64 64 64

needsupp_post 64 64 64 64 64 64 64

needsat_post 64 64 64 64 64 64 64

posaff_post 64 64 64 64 64 64 64

negaff_post 64 64 64 64 64 64 64

N

posaff_pre negaff_pre

needsat_post .079 .133

posaff_post .000 .487

negaff_post .168 .000

steps_post 64 64

AGwear_pre 64 64

AGwear_post 64 64

steps_pre 64 64

Weight_pre 64 64

needsupp_pre 64 64

needsat_pre 64 64

posaff_pre 64 64

negaff_pre 64 64

Age 64 64

Weight_post 64 64

needsupp_post 64 64

needsat_post 64 64

posaff_post 64 64

negaff_post 64 64

Age Weight_post needsupp_post needsat_post posaff_post

.208 .100 .000 . .000

.168 .422 .000 .000 .

.053 .047 .000 .001 .030

64 64 64 64 64

64 64 64 64 64

64 64 64 64 64

64 64 64 64 64

64 64 64 64 64

64 64 64 64 64

64 64 64 64 64

64 64 64 64 64

64 64 64 64 64

64 64 64 64 64

64 64 64 64 64

64 64 64 64 64

64 64 64 64 64

64 64 64 64 64

64 64 64 64 64

negaff_post

|  | needsat_post | .001 |
| --- | --- | --- |
|  | posaff_post | .030 |
|  | negaff_post | . |
| N | steps_post | 64 |
|  | AGwear_pre | 64 |
|  | AGwear_post | 64 |
|  | steps_pre | 64 |
|  | Weight_pre | 64 |
|  | needsupp_pre | 64 |
|  | needsat_pre | 64 |
|  | posaff_pre | 64 |
|  | negaff_pre | 64 |
|  | Age | 64 |
|  | Weight_post | 64 |
|  | needsupp_post | 64 |
|  | needsat_post | 64 |
|  | posaff_post | 64 |
|  | negaff_post | 64 |

## Variables Entered/Removeda

| Variables  Model Entered | | Variables Removed | Method |
| --- | --- | --- | --- |
| 1 | negaff_pre, steps_pre, AGwear_post, needsupp_pre, AGwear_pre, posaff_pre, Weight_pre,  needsat_preb | . | Enter |
| 2 | Age, Weight_postb | . | Enter |
| 3 | needsupp_postb | . | Enter |
| 4 | needsat_postb | . | Enter |
| 5 | posaff_post, negaff_postb | . | Enter |

1. Dependent Variable: steps_post
2. All requested variables entered.

## Model Summaryf

| Model R | | R Square | Adjusted R Square | Std. Error of the Estimate | Change Statistics | | | | |
| --- | --- | --- | --- | --- | --- | --- | --- | --- | --- |
|  |  |  |  |  | R Square Change | F Change | df1 | df2 | Sig. F Change |
| 1 | .728a | .530 | .461 | 3179.446 | .530 | 7.749 | 8 | 55 | <.001 |
| 2 | .789b | .623 | .552 | 2900.889 | .093 | 6.535 | 2 | 53 | .003 |
| 3 | .791c | .626 | .547 | 2917.107 | .003 | .412 | 1 | 52 | .524 |
| 4 | .792d | .628 | .540 | 2938.100 | .002 | .260 | 1 | 51 | .613 |
| 5 | .801e | .642 | .540 | 2938.974 | .014 | .985 | 2 | 49 | .381 |

1. Predictors: (Constant), negaff_pre, steps_pre, AGwear_post, needsupp_pre, AGwear_pre, posaff_pre, Weight_pre, needsat_pre
2. Predictors: (Constant), negaff_pre, steps_pre, AGwear_post, needsupp_pre, AGwear_pre, posaff_pre, Weight_pre, needsat_pre, Age,

Weight_post

1. Predictors: (Constant), negaff_pre, steps_pre, AGwear_post, needsupp_pre, AGwear_pre, posaff_pre, Weight_pre, needsat_pre, Age,

Weight_post, needsupp_post

1. Predictors: (Constant), negaff_pre, steps_pre, AGwear_post, needsupp_pre, AGwear_pre, posaff_pre, Weight_pre, needsat_pre, Age,

Weight_post, needsupp_post, needsat_post

1. Predictors: (Constant), negaff_pre, steps_pre, AGwear_post, needsupp_pre, AGwear_pre, posaff_pre, Weight_pre, needsat_pre, Age,

Weight_post, needsupp_post, needsat_post, posaff_post, negaff_post

1. Dependent Variable: steps_post

## ANOVAa

| Model Sum of Squares | | | df | Mean Square | F | Sig. |
| --- | --- | --- | --- | --- | --- | --- |
| 1 | Regression | 626634045.18 | 8 | 78329255.647 | 7.749 | <.001b |
|  | Residual | 555988130.80 | 55 | 10108875.105 |  |  |
|  | Total | 1182622176.0 | 63 |  |  |  |
| 2 | Regression | 736618849.20 | 10 | 73661884.920 | 8.753 | <.001c |
|  | Residual | 446003326.77 | 53 | 8415157.109 |  |  |
|  | Total | 1182622176.0 | 63 |  |  |  |
| 3 | Regression | 740127464.14 | 11 | 67284314.922 | 7.907 | <.001d |
|  | Residual | 442494711.83 | 52 | 8509513.689 |  |  |
|  | Total | 1182622176.0 | 63 |  |  |  |
| 4 | Regression | 742368221.73 | 12 | 61864018.477 | 7.166 | <.001e |
|  | Residual | 440253954.25 | 51 | 8632430.475 |  |  |
|  | Total | 1182622176.0 | 63 |  |  |  |
| 5 | Regression | 759381450.19 | 14 | 54241532.156 | 6.280 | <.001f |
|  | Residual | 423240725.79 | 49 | 8637565.832 |  |  |
|  | Total | 1182622176.0 | 63 |  |  |  |

1. Dependent Variable: steps_post
2. Predictors: (Constant), negaff_pre, steps_pre, AGwear_post, needsupp_pre,

AGwear_pre, posaff_pre, Weight_pre, needsat_pre

1. Predictors: (Constant), negaff_pre, steps_pre, AGwear_post, needsupp_pre,

AGwear_pre, posaff_pre, Weight_pre, needsat_pre, Age, Weight_post

1. Predictors: (Constant), negaff_pre, steps_pre, AGwear_post, needsupp_pre,

AGwear_pre, posaff_pre, Weight_pre, needsat_pre, Age, Weight_post, needsupp_post

1. Predictors: (Constant), negaff_pre, steps_pre, AGwear_post, needsupp_pre, AGwear_pre, posaff_pre, Weight_pre, needsat_pre, Age, Weight_post, needsupp_post, needsat_post
2. Predictors: (Constant), negaff_pre, steps_pre, AGwear_post, needsupp_pre, AGwear_pre, posaff_pre, Weight_pre, needsat_pre, Age, Weight_post, needsupp_post, needsat_post, posaff_post, negaff_post

## Coefficientsa

| Unstandardized Coefficients | | | | Standardized Coefficients |  |  | 95.0% Confidence Interval for B | | Correlations |
| --- | --- | --- | --- | --- | --- | --- | --- | --- | --- |
| Model |  | B | Std. Error | Beta | t | Sig. | Lower Bound | Upper Bound | Zero-order |
| 1 | (Constant) | -4507.613 | 5801.476 |  | -.777 | .441 | -16134.031 | 7118.805 |  |
|  | AGwear_pre | 4.456 | 3.438 | .138 | 1.296 | .200 | -2.434 | 11.346 | .300 |
|  | AGwear_post | 1.620 | 2.660 | .061 | .609 | .545 | -3.710 | 6.950 | .075 |
|  | steps_pre | .666 | .132 | .595 | 5.050 | <.001 | .402 | .930 | .683 |
|  | Weight_pre | -2.365 | 26.610 | -.010 | -.089 | .930 | -55.693 | 50.963 | -.318 |
|  | needsupp_pre | 639.605 | 407.862 | .183 | 1.568 | .123 | -177.768 | 1456.978 | .266 |
|  | needsat_pre | -373.551 | 851.999 | -.052 | -.438 | .663 | -2080.995 | 1333.894 | .080 |
|  | posaff_pre | 735.876 | 825.909 | .099 | .891 | .377 | -919.282 | 2391.034 | .283 |
|  | negaff_pre | 421.515 | 760.060 | .060 | .555 | .581 | -1101.678 | 1944.709 | .027 |
| 2 | (Constant) | -5022.506 | 5792.828 |  | -.867 | .390 | -16641.448 | 6596.435 |  |
|  | AGwear_pre | 3.172 | 3.157 | .098 | 1.005 | .320 | -3.160 | 9.504 | .300 |
|  | AGwear_post | 2.477 | 2.455 | .093 | 1.009 | .317 | -2.447 | 7.401 | .075 |
|  | steps_pre | .667 | .120 | .596 | 5.542 | <.001 | .426 | .908 | .683 |
|  | Weight_pre | 238.951 | 75.550 | 1.039 | 3.163 | .003 | 87.416 | 390.486 | -.318 |
|  | needsupp_pre | 569.886 | 385.761 | .163 | 1.477 | .146 | -203.853 | 1343.626 | .266 |
|  | needsat_pre | 131.428 | 810.382 | .018 | .162 | .872 | -1493.992 | 1756.849 | .080 |
|  | posaff_pre | 226.086 | 769.254 | .030 | .294 | .770 | -1316.842 | 1769.015 | .283 |
|  | negaff_pre | 1017.270 | 729.107 | .145 | 1.395 | .169 | -445.134 | 2479.674 | .027 |
|  | Age | 38.256 | 53.574 | .066 | .714 | .478 | -69.201 | 145.712 | .196 |
|  | Weight_post | -264.360 | 80.522 | -1.108 | -3.283 | .002 | -425.866 | -102.854 | -.397 |
| 3 | (Constant) | -4911.909 | 5827.760 |  | -.843 | .403 | -16606.165 | 6782.346 |  |
|  | AGwear_pre | 3.348 | 3.187 | .104 | 1.051 | .298 | -3.046 | 9.743 | .300 |

| Correlations | | | | Collinearity Statistics | |
| --- | --- | --- | --- | --- | --- |
| Model | | Partial | Part | Tolerance | VIF |
| 1 | (Constant) |  |  |  |  |
|  | AGwear_pre | .172 | .120 | .752 | 1.330 |
|  | AGwear_post | .082 | .056 | .866 | 1.155 |
|  | steps_pre | .563 | .467 | .616 | 1.625 |
|  | Weight_pre | -.012 | -.008 | .638 | 1.567 |
|  | needsupp_pre | .207 | .145 | .624 | 1.601 |
|  | needsat_pre | -.059 | -.041 | .597 | 1.676 |
|  | posaff_pre | .119 | .082 | .697 | 1.435 |
|  | negaff_pre | .075 | .051 | .725 | 1.380 |
| 2 | (Constant) |  |  |  |  |
|  | AGwear_pre | .137 | .085 | .742 | 1.347 |
|  | AGwear_post | .137 | .085 | .846 | 1.182 |
|  | steps_pre | .606 | .468 | .615 | 1.625 |
|  | Weight_pre | .398 | .267 | .066 | 15.175 |
|  | needsupp_pre | .199 | .125 | .581 | 1.721 |
|  | needsat_pre | .022 | .014 | .549 | 1.821 |
|  | posaff_pre | .040 | .025 | .669 | 1.496 |
|  | negaff_pre | .188 | .118 | .655 | 1.526 |
|  | Age | .098 | .060 | .828 | 1.208 |
|  | Weight_post | -.411 | -.277 | .063 | 15.992 |
| 3 | (Constant) |  |  |  |  |
|  | AGwear_pre | .144 | .089 | .737 | 1.357 |

| Unstandardized Coefficients | | | | Standardized Coefficients |  |  | 95.0% Confidence Interval for B | | Correlations |
| --- | --- | --- | --- | --- | --- | --- | --- | --- | --- |
| Model |  | B | Std. Error | Beta | t | Sig. | Lower Bound | Upper Bound | Zero-order |
|  | AGwear_post | 2.947 | 2.574 | .110 | 1.145 | .258 | -2.220 | 8.113 | .075 |
|  | steps_pre | .667 | .121 | .596 | 5.514 | <.001 | .425 | .910 | .683 |
|  | Weight_pre | 278.217 | 97.526 | 1.210 | 2.853 | .006 | 82.517 | 473.916 | -.318 |
|  | needsupp_pre | 724.844 | 456.855 | .208 | 1.587 | .119 | -191.903 | 1641.590 | .266 |
|  | needsat_pre | 139.442 | 815.008 | .020 | .171 | .865 | -1495.991 | 1774.876 | .080 |
|  | posaff_pre | 224.894 | 773.557 | .030 | .291 | .772 | -1327.362 | 1777.150 | .283 |
|  | negaff_pre | 983.474 | 735.070 | .141 | 1.338 | .187 | -491.552 | 2458.501 | .027 |
|  | Age | 38.846 | 53.882 | .067 | .721 | .474 | -69.275 | 146.968 | .196 |
|  | Weight_post | -300.710 | 98.798 | -1.260 | -3.044 | .004 | -498.963 | -102.457 | -.397 |
|  | needsupp_post | -350.914 | 546.494 | -.091 | -.642 | .524 | -1447.535 | 745.706 | .200 |
| 4 | (Constant) | -5575.984 | 6012.677 |  | -.927 | .358 | -17646.937 | 6494.968 |  |
|  | AGwear_pre | 3.142 | 3.235 | .097 | .971 | .336 | -3.352 | 9.637 | .300 |
|  | AGwear_post | 2.952 | 2.593 | .110 | 1.138 | .260 | -2.254 | 8.157 | .075 |
|  | steps_pre | .672 | .122 | .600 | 5.497 | <.001 | .426 | .917 | .683 |
|  | Weight_pre | 263.312 | 102.491 | 1.145 | 2.569 | .013 | 57.552 | 469.072 | -.318 |
|  | needsupp_pre | 782.524 | 473.866 | .224 | 1.651 | .105 | -168.801 | 1733.849 | .266 |
|  | needsat_pre | 38.778 | 844.317 | .005 | .046 | .964 | -1656.258 | 1733.815 | .080 |
|  | posaff_pre | 192.252 | 781.754 | .026 | .246 | .807 | -1377.184 | 1761.689 | .283 |
|  | negaff_pre | 965.870 | 741.166 | .138 | 1.303 | .198 | -522.083 | 2453.823 | .027 |
|  | Age | 44.793 | 55.510 | .078 | .807 | .423 | -66.649 | 156.234 | .196 |
|  | Weight_post | -286.837 | 103.167 | -1.202 | -2.780 | .008 | -493.954 | -79.720 | -.397 |
|  | needsupp_post | -476.130 | 602.804 | -.124 | -.790 | .433 | -1686.309 | 734.049 | .200 |
|  | needsat_post | 394.099 | 773.526 | .062 | .509 | .613 | -1158.819 | 1947.018 | .100 |
| 5 | (Constant) | -5162.954 | 6916.177 |  | -.747 | .459 | -19061.532 | 8735.624 |  |
|  | AGwear_pre | 3.404 | 3.268 | .106 | 1.042 | .303 | -3.163 | 9.971 | .300 |
|  | AGwear_post | 3.552 | 2.629 | .133 | 1.351 | .183 | -1.732 | 8.836 | .075 |

| Correlations | | | | Collinearity Statistics | |
| --- | --- | --- | --- | --- | --- |
| Model | | Partial | Part | Tolerance | VIF |
|  | AGwear_post | .157 | .097 | .778 | 1.285 |
|  | steps_pre | .607 | .468 | .615 | 1.625 |
|  | Weight_pre | .368 | .242 | .040 | 25.007 |
|  | needsupp_pre | .215 | .135 | .419 | 2.387 |
|  | needsat_pre | .024 | .015 | .549 | 1.822 |
|  | posaff_pre | .040 | .025 | .669 | 1.496 |
|  | negaff_pre | .182 | .113 | .652 | 1.533 |
|  | Age | .099 | .061 | .827 | 1.208 |
|  | Weight_post | -.389 | -.258 | .042 | 23.809 |
|  | needsupp_post | -.089 | -.054 | .358 | 2.796 |
| 4 | (Constant) |  |  |  |  |
|  | AGwear_pre | .135 | .083 | .725 | 1.379 |
|  | AGwear_post | .157 | .097 | .778 | 1.285 |
|  | steps_pre | .610 | .470 | .613 | 1.632 |
|  | Weight_pre | .339 | .219 | .037 | 27.225 |
|  | needsupp_pre | .225 | .141 | .395 | 2.531 |
|  | needsat_pre | .006 | .004 | .519 | 1.927 |
|  | posaff_pre | .034 | .021 | .664 | 1.506 |
|  | negaff_pre | .180 | .111 | .651 | 1.537 |
|  | Age | .112 | .069 | .791 | 1.264 |
|  | Weight_post | -.363 | -.238 | .039 | 25.592 |
|  | needsupp_post | -.110 | -.067 | .298 | 3.354 |
|  | needsat_post | .071 | .044 | .486 | 2.056 |
| 5 | (Constant) |  |  |  |  |
|  | AGwear_pre | .147 | .089 | .711 | 1.406 |
|  | AGwear_post | .189 | .115 | .757 | 1.321 |

| Unstandardized Coefficients | | | | Standardized Coefficients |  |  | 95.0% Confidence Interval for B | | Correlations |
| --- | --- | --- | --- | --- | --- | --- | --- | --- | --- |
| Model |  | B | Std. Error | Beta | t | Sig. | Lower Bound | Upper Bound | Zero-order |
|  | steps_pre | .680 | .122 | .608 | 5.553 | <.001 | .434 | .926 | .683 |
|  | Weight_pre | 279.220 | 103.202 | 1.214 | 2.706 | .009 | 71.828 | 486.613 | -.318 |
|  | needsupp_pre | 708.020 | 481.114 | .203 | 1.472 | .148 | -258.815 | 1674.855 | .266 |
|  | needsat_pre | -8.123 | 847.472 | -.001 | -.010 | .992 | -1711.182 | 1694.935 | .080 |
|  | posaff_pre | 697.620 | 870.794 | .094 | .801 | .427 | -1052.305 | 2447.545 | .283 |
|  | negaff_pre | 1261.838 | 991.343 | .180 | 1.273 | .209 | -730.340 | 3254.016 | .027 |
|  | Age | 45.398 | 59.402 | .079 | .764 | .448 | -73.974 | 164.771 | .196 |
|  | Weight_post | -306.787 | 104.210 | -1.285 | -2.944 | .005 | -516.206 | -97.369 | -.397 |
|  | needsupp_post | -386.544 | 637.442 | -.100 | -.606 | .547 | -1667.532 | 894.443 | .200 |
|  | needsat_post | 788.725 | 844.218 | .125 | .934 | .355 | -907.795 | 2485.245 | .100 |
|  | posaff_post | -1186.252 | 846.866 | -.174 | -1.401 | .168 | -2888.094 | 515.590 | .192 |
|  | negaff_post | -193.716 | 1324.910 | -.022 | -.146 | .884 | -2856.221 | 2468.790 | -.100 |

| Correlations | | | | Collinearity Statistics | |
| --- | --- | --- | --- | --- | --- |
| Model | | Partial | Part | Tolerance | VIF |
|  | steps_pre | .621 | .475 | .610 | 1.640 |
|  | Weight_pre | .361 | .231 | .036 | 27.587 |
|  | needsupp_pre | .206 | .126 | .383 | 2.608 |
|  | needsat_pre | -.001 | -.001 | .515 | 1.941 |
|  | posaff_pre | .114 | .068 | .536 | 1.867 |
|  | negaff_pre | .179 | .109 | .364 | 2.748 |
|  | Age | .109 | .065 | .691 | 1.447 |
|  | Weight_post | -.388 | -.252 | .038 | 26.096 |
|  | needsupp_post | -.086 | -.052 | .267 | 3.748 |
|  | needsat_post | .132 | .080 | .409 | 2.447 |
|  | posaff_post | -.196 | -.120 | .471 | 2.122 |
|  | negaff_post | -.021 | -.012 | .332 | 3.016 |

1. Dependent Variable: steps_post

## Excluded Variablesa

| Model Beta In | | | t | Sig. | Partial Correlation | Collinearity Statistics | | |
| --- | --- | --- | --- | --- | --- | --- | --- | --- |
|  |  |  |  |  |  | Tolerance | VIF | Minimum Tolerance |
| 1 | Age | .137b | 1.393 | .169 | .186 | .874 | 1.144 | .586 |
|  | Weight_post | -1.163b | -3.560 | <.001 | -.436 | .066 | 15.138 | .066 |
|  | needsupp_post | .179b | 1.439 | .156 | .192 | .544 | 1.837 | .467 |
|  | needsat_post | .169b | 1.643 | .106 | .218 | .788 | 1.269 | .576 |
|  | posaff_post | .036b | .325 | .746 | .044 | .727 | 1.375 | .576 |
|  | negaff_post | -.159b | -1.245 | .219 | -.167 | .517 | 1.935 | .445 |
| 2 | needsupp_post | -.091c | -.642 | .524 | -.089 | .358 | 2.796 | .040 |
|  | needsat_post | .023c | .206 | .838 | .029 | .583 | 1.714 | .045 |
|  | posaff_post | -.139c | -1.279 | .207 | -.175 | .592 | 1.688 | .051 |
|  | negaff_post | .010c | .078 | .938 | .011 | .414 | 2.413 | .057 |
| 3 | needsat_post | .062d | .509 | .613 | .071 | .486 | 2.056 | .037 |
|  | posaff_post | -.130d | -1.130 | .264 | -.156 | .543 | 1.843 | .038 |
|  | negaff_post | -.028d | -.193 | .847 | -.027 | .348 | 2.873 | .040 |
| 4 | posaff_post | -.174e | -1.410 | .165 | -.196 | .472 | 2.118 | .036 |
|  | negaff_post | -.013e | -.086 | .932 | -.012 | .332 | 3.010 | .037 |

1. Dependent Variable: steps_post
2. Predictors in the Model: (Constant), negaff_pre, steps_pre, AGwear_post, needsupp_pre, AGwear_pre, posaff_pre,

Weight_pre, needsat_pre

1. Predictors in the Model: (Constant), negaff_pre, steps_pre, AGwear_post, needsupp_pre, AGwear_pre, posaff_pre,

Weight_pre, needsat_pre, Age, Weight_post

1. Predictors in the Model: (Constant), negaff_pre, steps_pre, AGwear_post, needsupp_pre, AGwear_pre, posaff_pre,

Weight_pre, needsat_pre, Age, Weight_post, needsupp_post

1. Predictors in the Model: (Constant), negaff_pre, steps_pre, AGwear_post, needsupp_pre, AGwear_pre, posaff_pre, Weight_pre, needsat_pre, Age, Weight_post, needsupp_post, needsat_post

Model Dimension Eigenvalue

Condition Index

(Constant)

AGwear_pre

AGwear_post

steps_pre

Weight_pre

needsupp_pre

1 1 8.648

2 .140

3 .084

4 .042

5 .034

6 .018

7 .018

8 .014

9 .004

2 1 10.593

2 .145

3 .099

4 .046

5 .035

6 .029

7 .021

8 .016

9 .012

10 .003

11 .001

3 1 11.561

2 .150

3 .103

4 .046

5 .038

6 .030

1.000

7.869

10.171

14.372

15.989

22.123

22.196

25.270

47.735

1.000

8.559

10.368

15.174

17.336

19.078

22.268

26.122

29.113

55.770

108.649

1.000

8.781

10.604

15.772

17.408

19.774

.00

.00

.00

.00

.00

.00

.01

.01

.98

.00

.00

.00

.00

.00

.00

.00

.00

.00

.99

.01

.00

.00

.00

.00

.00

.00

.00

.00

.00

.04

.10

.00

.00

.55

.32

.00

.00

.00

.02

.12

.00

.00

.05

.56

.24

.01

.00

.00

.00

.01

.09

.00

.00

.00

.01

.25

.00

.01

.19

.52

.02

.00

.00

.00

.14

.04

.06

.27

.17

.29

.01

.01

.00

.00

.00

.12

.05

.03

.00

.01

.42

.00

.00

.05

.25

.17

.10

.00

.02

.28

.01

.03

.26

.21

.03

.07

.08

.01

.00

.01

.28

.01

.01

.26

.00

.00

.04

.00

.02

.20

.34

.01

.39

.00

.00

.00

.00

.00

.01

.01

.00

.00

.02

.97

.00

.00

.00

.00

.00

.00

.00

.03

.01

.23

.21

.25

.19

.07

.02

.00

.03

.01

.23

.16

.00

.04

.20

.31

.00

.03

.00

.02

.00

.17

.04

.02

| Model Dimension | | needsat_pre | posaff_pre | negaff_pre | Age | Weight_post | needsupp_post | needsat_post | posaff_post | negaff_post |
| --- | --- | --- | --- | --- | --- | --- | --- | --- | --- | --- |
| 1 | 1 | .00 | .00 | .00 |  |  |  |  |  |  |
|  | 2 | .01 | .01 | .42 |  |  |  |  |  |  |
|  | 3 | .01 | .00 | .01 |  |  |  |  |  |  |
|  | 4 | .03 | .00 | .22 |  |  |  |  |  |  |
|  | 5 | .00 | .43 | .00 |  |  |  |  |  |  |
|  | 6 | .69 | .02 | .09 |  |  |  |  |  |  |
|  | 7 | .06 | .30 | .24 |  |  |  |  |  |  |
|  | 8 | .01 | .23 | .02 |  |  |  |  |  |  |
|  | 9 | .18 | .01 | .01 |  |  |  |  |  |  |
| 2 | 1 | .00 | .00 | .00 | .00 | .00 |  |  |  |  |
|  | 2 | .01 | .01 | .31 | .00 | .00 |  |  |  |  |
|  | 3 | .01 | .00 | .07 | .00 | .00 |  |  |  |  |
|  | 4 | .01 | .00 | .17 | .09 | .00 |  |  |  |  |
|  | 5 | .00 | .31 | .00 | .01 | .00 |  |  |  |  |
|  | 6 | .09 | .00 | .12 | .30 | .01 |  |  |  |  |
|  | 7 | .01 | .38 | .22 | .09 | .01 |  |  |  |  |
|  | 8 | .63 | .13 | .05 | .13 | .00 |  |  |  |  |
|  | 9 | .08 | .15 | .01 | .18 | .00 |  |  |  |  |
|  | 10 | .09 | .00 | .00 | .15 | .01 |  |  |  |  |
|  | 11 | .07 | .03 | .06 | .05 | .98 |  |  |  |  |
| 3 | 1 | .00 | .00 | .00 | .00 | .00 | .00 |  |  |  |
|  | 2 | .01 | .01 | .31 | .00 | .00 | .00 |  |  |  |
|  | 3 | .00 | .00 | .03 | .00 | .00 | .01 |  |  |  |
|  | 4 | .01 | .00 | .19 | .09 | .00 | .01 |  |  |  |
|  | 5 | .03 | .24 | .00 | .00 | .00 | .05 |  |  |  |
|  | 6 | .13 | .01 | .09 | .24 | .00 | .01 |  |  |  |

Model Dimension Eigenvalue

Condition Index

(Constant)

AGwear_pre

AGwear_post

steps_pre

Weight_pre

needsupp_pre

7 .023

8 .021

9 .013

10 .011

11 .003

12 .001

4 1 12.536

2 .151

3 .107

4 .046

5 .039

6 .031

7 .026

8 .022

9 .017

10 .013

11 .007

12 .003

13 .001

5 1 14.413

2 .213

3 .109

4 .060

5 .044

6 .037

7 .031

22.387

23.631

29.333

32.506

58.347

143.531

1.000

9.115

10.820

16.420

17.867

20.049

21.953

24.134

27.107

30.548

41.771

61.959

155.332

1.000

8.233

11.495

15.443

18.164

19.713

21.730

.00

.00

.00

.00

.99

.00

.00

.00

.00

.00

.00

.00

.00

.00

.00

.00

.00

.97

.02

.00

.00

.00

.00

.00

.00

.00

.03

.00

.52

.10

.25

.00

.00

.00

.00

.01

.07

.02

.02

.00

.02

.52

.15

.19

.00

.00

.00

.00

.01

.00

.08

.02

.00

.34

.29

.09

.01

.07

.00

.00

.00

.12

.03

.00

.11

.29

.00

.29

.06

.02

.06

.00

.00

.00

.02

.04

.07

.12

.10

.12

.12

.00

.08

.00

.00

.00

.27

.01

.00

.23

.08

.18

.01

.12

.00

.09

.00

.00

.00

.26

.00

.01

.02

.06

.00

.00

.00

.00

.01

.98

.00

.00

.00

.00

.00

.00

.00

.00

.00

.00

.00

.01

.98

.00

.00

.00

.00

.00

.00

.00

.00

.02

.01

.67

.00

.05

.00

.02

.00

.16

.01

.07

.02

.03

.10

.00

.54

.02

.02

.00

.01

.00

.08

.10

.02

.00

| Model Dimension | | needsat_pre | posaff_pre | negaff_pre | Age | Weight_post | needsupp_post | needsat_post | posaff_post | negaff_post |
| --- | --- | --- | --- | --- | --- | --- | --- | --- | --- | --- |
|  | 7 | .13 | .29 | .13 | .03 | .00 | .18 |  |  |  |
|  | 8 | .04 | .14 | .13 | .23 | .00 | .07 |  |  |  |
|  | 9 | .13 | .28 | .06 | .01 | .00 | .06 |  |  |  |
|  | 10 | .41 | .00 | .03 | .21 | .00 | .22 |  |  |  |
|  | 11 | .08 | .00 | .00 | .16 | .01 | .00 |  |  |  |
|  | 12 | .04 | .02 | .02 | .02 | .98 | .39 |  |  |  |
| 4 | 1 | .00 | .00 | .00 | .00 | .00 | .00 | .00 |  |  |
|  | 2 | .01 | .01 | .32 | .00 | .00 | .00 | .00 |  |  |
|  | 3 | .00 | .00 | .02 | .00 | .00 | .00 | .01 |  |  |
|  | 4 | .01 | .00 | .19 | .09 | .00 | .00 | .00 |  |  |
|  | 5 | .05 | .21 | .00 | .01 | .00 | .05 | .02 |  |  |
|  | 6 | .10 | .06 | .03 | .13 | .00 | .02 | .06 |  |  |
|  | 7 | .00 | .06 | .12 | .15 | .01 | .05 | .09 |  |  |
|  | 8 | .02 | .36 | .18 | .07 | .00 | .00 | .02 |  |  |
|  | 9 | .32 | .00 | .04 | .21 | .00 | .06 | .21 |  |  |
|  | 10 | .11 | .28 | .06 | .01 | .00 | .05 | .00 |  |  |
|  | 11 | .28 | .02 | .01 | .09 | .00 | .54 | .46 |  |  |
|  | 12 | .04 | .00 | .00 | .19 | .00 | .04 | .05 |  |  |
|  | 13 | .07 | .01 | .02 | .04 | .98 | .18 | .08 |  |  |
| 5 | 1 | .00 | .00 | .00 | .00 | .00 | .00 | .00 | .00 | .00 |
|  | 2 | .00 | .00 | .08 | .00 | .00 | .00 | .00 | .00 | .05 |
|  | 3 | .00 | .00 | .00 | .00 | .00 | .00 | .00 | .00 | .00 |
|  | 4 | .04 | .01 | .04 | .02 | .00 | .00 | .01 | .02 | .11 |
|  | 5 | .01 | .06 | .17 | .05 | .00 | .03 | .01 | .00 | .02 |
|  | 6 | .01 | .15 | .01 | .04 | .00 | .00 | .02 | .07 | .00 |
|  | 7 | .08 | .01 | .18 | .21 | .00 | .00 | .00 | .00 | .08 |

Model Dimension Eigenvalue Condition Index (Constant) AGwear_pre AGwear_post steps_pre Weight_pre needsupp_pre

8 .026 23.582 .00 .01 .04 .47 .01 .02

9 .019 27.434 .00 .04 .25 .00 .00 .07

10 .016 29.642 .00 .00 .13 .01 .00 .03

11 .013 33.892 .00 .55 .17 .07 .00 .01

12 .010 38.648 .00 .01 .00 .01 .00 .09

13 .007 45.399 .00 .09 .07 .00 .00 .54

14 .002 76.302 .97 .19 .02 .08 .01 .00

15 .001 167.991 .02 .00 .07 .00 .98 .01

## Collinearity Diagnosticsa

Variance Proportions

| Model Dimension | | needsat_pre | posaff_pre | negaff_pre | Age | Weight_post | needsupp_post | needsat_post | posaff_post | negaff_post |
| --- | --- | --- | --- | --- | --- | --- | --- | --- | --- | --- |
|  | 8 | .00 | .03 | .00 | .00 | .01 | .00 | .00 | .07 | .10 |
|  | 9 | .05 | .15 | .15 | .00 | .00 | .01 | .17 | .01 | .11 |
|  | 10 | .42 | .00 | .18 | .23 | .00 | .06 | .02 | .00 | .10 |
|  | 11 | .02 | .23 | .01 | .05 | .00 | .10 | .02 | .00 | .11 |
|  | 12 | .00 | .35 | .04 | .00 | .00 | .06 | .22 | .80 | .00 |
|  | 13 | .26 | .00 | .00 | .07 | .00 | .56 | .41 | .01 | .02 |
|  | 14 | .06 | .01 | .11 | .28 | .00 | .00 | .07 | .00 | .27 |
|  | 15 | .06 | .00 | .03 | .05 | .98 | .16 | .05 | .01 | .00 |

1. Dependent Variable: steps_post

## Residuals Statisticsa

| Minimum | | Maximum | Mean | Std. Deviation | N |
| --- | --- | --- | --- | --- | --- |
| Predicted Value | 7560.36 | 24529.79 | 14410.37 | 3471.840 | 64 |
| Std. Predicted Value | -1.973 | 2.915 | .000 | 1.000 | 64 |
| Standard Error of Predicted Value | 827.858 | 2061.565 | 1393.468 | 289.810 | 64 |
| Adjusted Predicted Value | 7349.80 | 24846.28 | 14419.85 | 3587.762 | 64 |
| Residual | -5087.346 | 5645.471 | .000 | 2591.931 | 64 |
| Std. Residual | -1.731 | 1.921 | .000 | .882 | 64 |
| Stud. Residual | -1.996 | 2.387 | -.001 | 1.006 | 64 |
| Deleted Residual | -7068.854 | 8714.535 | -9.475 | 3405.065 | 64 |
| Stud. Deleted Residual | -2.062 | 2.513 | -.001 | 1.023 | 64 |
| Mahal. Distance | 4.014 | 30.014 | 13.781 | 6.053 | 64 |
| Cook's Distance | .000 | .206 | .022 | .037 | 64 |
| Centered Leverage Value | .064 | .476 | .219 | .096 | 64 |

1. Dependent Variable: steps_post

**Charts**

# Normal P-P Plot of Regression Standardized Residual Dependent Variable: steps_post

1.0

0.8

**Expected Cum Prob**

0.6

0.4

0.2

0.0

0.0

0.2

0.4

0.6

0.8

1.0

# Observed Cum Prob

**Scatterplot Dependent Variable: steps_post**

2

**Regression Standardized Residual**

1

0

-1

-2

-2 -1 0 1 2 3

# Regression Standardized Predicted Value

**Regression**

## Descriptive Statistics

| Mean | | Std. Deviation | N |
| --- | --- | --- | --- |
| MVPA_post | 45.67 | 21.588 | 64 |
| AGwear_pre | 924.55 | 134.352 | 64 |
| AGwear_post | 938.53 | 161.837 | 64 |
| MVPA_pre | 36.53 | 19.808 | 64 |
| Weight_pre | 110.84 | 18.845 | 64 |
| needsupp_pre | 5.16 | 1.243 | 64 |
| needsat_pre | 3.24 | .609 | 64 |
| posaff_pre | 2.83 | .581 | 64 |
| negaff_pre | 1.71 | .619 | 64 |
| Age | 45.0547 | 7.49821 | 64 |
| Weight_post | 107.59 | 18.151 | 64 |
| needsupp_post | 5.56 | 1.125 | 64 |
| needsat_post | 3.89 | .686 | 64 |
| posaff_post | 3.48 | .637 | 64 |
| negaff_post | 1.48 | .485 | 64 |

Pearson Correlation MVPA_post

AGwear_pre AGwear_post MVPA_pre Weight_pre needsupp_pre needsat_pre posaff_pre negaff_pre Age Weight_post needsupp_post needsat_post posaff_post negaff_post

Sig. (1-tailed) MVPA_post AGwear_pre AGwear_post MVPA_pre Weight_pre needsupp_pre needsat_pre posaff_pre negaff_pre Age Weight_post

needsupp_post

MVPA_post

1.000

.089

.032

.448

-.189

.284

.094

.102

-.076

.111

-.322

.358

.129

.164

-.170

.

.242

.400

.000

.068

.011

.230

.211

.274

.191

.005

.002

AGwear_pre

.089

1.000

.260

.094

-.233

.094

-.124

-.137

.102

.068

-.239

.113

.057

.124

-.099

.242

.

.019

.231

.032

.229

.164

.141

.210

.296

.029

.188

AGwear_post

.032

.260

1.000

-.111

.054

-.044

-.040

.091

-.053

.086

.065

.183

.110

.242

-.153

.400

.019

.

.192

.335

.364

.376

.238

.339

.250

.306

.074

MVPA_pre

.448

.094

-.111

1.000

-.320

.239

.200

.209

.015

.141

-.356

.005

-.197

.072

-.029

<.001

.231

.192

.

.005

.029

.056

.049

.452

.134

.002

.485

Weight_pre

-.189

-.233

.054

-.320

1.000

-.040

-.062

-.019

.229

-.200

.961

.229

.305

.092

.108

.068

.032

.335

.005

.

.377

.314

.440

.034

.056

.000

.035

needsupp_pre

.284

.094

-.044

.239

-.040

1.000

.545

.303

-.310

-.145

-.077

.560

.214

.109

-.266

.011

.229

.364

.029

.377

.

.000

.007

.006

.126

.273

.000

needsat_pre

.094

-.124

-.040

.200

-.062

.545

1.000

.383

-.378

.049

-.051

.252

.184

.081

-.299

.230

.164

.376

.056

.314

.000

.

.001

.001

.350

.346

.022

Pearson Correlation MVPA_post

AGwear_pre AGwear_post MVPA_pre Weight_pre needsupp_pre needsat_pre posaff_pre negaff_pre Age Weight_post needsupp_post needsat_post posaff_post negaff_post

Sig. (1-tailed) MVPA_post AGwear_pre AGwear_post MVPA_pre Weight_pre needsupp_pre needsat_pre posaff_pre negaff_pre Age Weight_post

needsupp_post

posaff_pre

.102

-.137

.091

.209

-.019

.303

.383

1.000

-.217

.130

-.062

.242

.178

.437

-.122

.211

.141

.238

.049

.440

.007

.001

.

.042

.153

.313

.027

negaff_pre

-.076

.102

-.053

.015

.229

-.310

-.378

-.217

1.000

.044

.286

-.261

-.141

-.004

.660

.274

.210

.339

.452

.034

.006

.001

.042

.

.364

.011

.019

Age

.111

.068

.086

.141

-.200

-.145

.049

.130

.044

1.000

-.236

-.021

-.103

.122

-.204

.191

.296

.250

.134

.056

.126

.350

.153

.364

.

.030

.436

Weight_post

-.322

-.239

.065

-.356

.961

-.077

-.051

-.062

.286

-.236

1.000

.071

.162

-.025

.212

.005

.029

.306

.002

.000

.273

.346

.313

.011

.030

.

.290

needsupp_post

.358

.113

.183

.005

.229

.560

.252

.242

-.261

-.021

.071

1.000

.624

.457

-.511

.002

.188

.074

.485

.035

.000

.022

.027

.019

.436

.290

.

needsat_post

.129

.057

.110

-.197

.305

.214

.184

.178

-.141

-.103

.162

.624

1.000

.546

-.395

.155

.326

.194

.060

.007

.045

.073

.079

.133

.208

.100

.000

posaff_post

.164

.124

.242

.072

.092

.109

.081

.437

-.004

.122

-.025

.457

.546

1.000

-.236

.097

.164

.027

.285

.236

.195

.262

.000

.487

.168

.422

.000

negaff_post

| Pearson Correlation | MVPA_post | -.170 |
| --- | --- | --- |
|  | AGwear_pre | -.099 |
|  | AGwear_post | -.153 |
|  | MVPA_pre | -.029 |
|  | Weight_pre | .108 |
|  | needsupp_pre | -.266 |
|  | needsat_pre | -.299 |
|  | posaff_pre | -.122 |
|  | negaff_pre | .660 |
|  | Age | -.204 |
|  | Weight_post | .212 |
|  | needsupp_post | -.511 |
|  | needsat_post | -.395 |
|  | posaff_post | -.236 |
|  | negaff_post | 1.000 |
| Sig. (1-tailed) | MVPA_post | .089 |
|  | AGwear_pre | .219 |
|  | AGwear_post | .114 |
|  | MVPA_pre | .410 |
|  | Weight_pre | .198 |
|  | needsupp_pre | .017 |
|  | needsat_pre | .008 |
|  | posaff_pre | .168 |
|  | negaff_pre | .000 |
|  | Age | .053 |
|  | Weight_post | .047 |
|  | needsupp_post | .000 |

N

MVPA_post AGwear_pre AGwear_post MVPA_pre Weight_pre needsupp_pre needsat_pre needsat_post .155 .326 .194 .060 .007 .045 .073

posaff_post .097 .164 .027 .285 .236 .195 .262

negaff_post .089 .219 .114 .410 .198 .017 .008

MVPA_post 64 64 64 64 64 64 64

AGwear_pre 64 64 64 64 64 64 64

AGwear_post 64 64 64 64 64 64 64

MVPA_pre 64 64 64 64 64 64 64

Weight_pre 64 64 64 64 64 64 64

needsupp_pre 64 64 64 64 64 64 64

needsat_pre 64 64 64 64 64 64 64

posaff_pre 64 64 64 64 64 64 64

negaff_pre 64 64 64 64 64 64 64

Age 64 64 64 64 64 64 64

Weight_post 64 64 64 64 64 64 64

needsupp_post 64 64 64 64 64 64 64

needsat_post 64 64 64 64 64 64 64

posaff_post 64 64 64 64 64 64 64

negaff_post 64 64 64 64 64 64 64

N

posaff_pre negaff_pre

needsat_post .079 .133

posaff_post .000 .487

negaff_post .168 .000

MVPA_post 64 64

AGwear_pre 64 64

AGwear_post 64 64

MVPA_pre 64 64

Weight_pre 64 64

needsupp_pre 64 64

needsat_pre 64 64

posaff_pre 64 64

negaff_pre 64 64

Age 64 64

Weight_post 64 64

needsupp_post 64 64

needsat_post 64 64

posaff_post 64 64

negaff_post 64 64

Age Weight_post needsupp_post needsat_post posaff_post

.208 .100 .000 . .000

.168 .422 .000 .000 .

.053 .047 .000 .001 .030

64 64 64 64 64

64 64 64 64 64

64 64 64 64 64

64 64 64 64 64

64 64 64 64 64

64 64 64 64 64

64 64 64 64 64

64 64 64 64 64

64 64 64 64 64

64 64 64 64 64

64 64 64 64 64

64 64 64 64 64

64 64 64 64 64

64 64 64 64 64

64 64 64 64 64

negaff_post

|  | needsat_post | .001 |
| --- | --- | --- |
|  | posaff_post | .030 |
|  | negaff_post | . |
| N | MVPA_post | 64 |
|  | AGwear_pre | 64 |
|  | AGwear_post | 64 |
|  | MVPA_pre | 64 |
|  | Weight_pre | 64 |
|  | needsupp_pre | 64 |
|  | needsat_pre | 64 |
|  | posaff_pre | 64 |
|  | negaff_pre | 64 |
|  | Age | 64 |
|  | Weight_post | 64 |
|  | needsupp_post | 64 |
|  | needsat_post | 64 |
|  | posaff_post | 64 |
|  | negaff_post | 64 |

## Variables Entered/Removeda

| Variables  Model Entered | | Variables Removed | Method |
| --- | --- | --- | --- |
| 1 | negaff_pre, MVPA_pre, AGwear_post, AGwear_pre, posaff_pre, needsupp_pre, Weight_pre,  needsat_preb | . | Enter |
| 2 | Age, Weight_postb | . | Enter |
| 3 | needsupp_postb | . | Enter |
| 4 | needsat_postb | . | Enter |
| 5 | posaff_post, negaff_postb | . | Enter |

1. Dependent Variable: MVPA_post
2. All requested variables entered.

## Model Summaryf

| Model R | | R Square | Adjusted R Square | Std. Error of the Estimate | Change Statistics | | | | |
| --- | --- | --- | --- | --- | --- | --- | --- | --- | --- |
|  |  |  |  |  | R Square Change | F Change | df1 | df2 | Sig. F Change |
| 1 | .509a | .259 | .151 | 19.894 | .259 | 2.398 | 8 | 55 | .027 |
| 2 | .669b | .448 | .344 | 17.484 | .190 | 9.104 | 2 | 53 | <.001 |
| 3 | .674c | .454 | .339 | 17.552 | .006 | .589 | 1 | 52 | .446 |
| 4 | .676d | .458 | .330 | 17.670 | .003 | .308 | 1 | 51 | .581 |
| 5 | .681e | .464 | .311 | 17.917 | .007 | .301 | 2 | 49 | .741 |

1. Predictors: (Constant), negaff_pre, MVPA_pre, AGwear_post, AGwear_pre, posaff_pre, needsupp_pre, Weight_pre, needsat_pre
2. Predictors: (Constant), negaff_pre, MVPA_pre, AGwear_post, AGwear_pre, posaff_pre, needsupp_pre, Weight_pre, needsat_pre, Age,

Weight_post

1. Predictors: (Constant), negaff_pre, MVPA_pre, AGwear_post, AGwear_pre, posaff_pre, needsupp_pre, Weight_pre, needsat_pre, Age,

Weight_post, needsupp_post

1. Predictors: (Constant), negaff_pre, MVPA_pre, AGwear_post, AGwear_pre, posaff_pre, needsupp_pre, Weight_pre, needsat_pre, Age,

Weight_post, needsupp_post, needsat_post

1. Predictors: (Constant), negaff_pre, MVPA_pre, AGwear_post, AGwear_pre, posaff_pre, needsupp_pre, Weight_pre, needsat_pre, Age,

Weight_post, needsupp_post, needsat_post, posaff_post, negaff_post

1. Dependent Variable: MVPA_post

## ANOVAa

| Model Sum of Squares | | | df | Mean Square | F | Sig. |
| --- | --- | --- | --- | --- | --- | --- |
| 1 | Regression | 7592.420 | 8 | 949.053 | 2.398 | .027b |
|  | Residual | 21767.536 | 55 | 395.773 |  |  |
|  | Total | 29359.956 | 63 |  |  |  |
| 2 | Regression | 13158.495 | 10 | 1315.850 | 4.305 | <.001c |
|  | Residual | 16201.461 | 53 | 305.688 |  |  |
|  | Total | 29359.956 | 63 |  |  |  |
| 3 | Regression | 13339.815 | 11 | 1212.710 | 3.936 | <.001d |
|  | Residual | 16020.141 | 52 | 308.080 |  |  |
|  | Total | 29359.956 | 63 |  |  |  |
| 4 | Regression | 13435.939 | 12 | 1119.662 | 3.586 | <.001e |
|  | Residual | 15924.017 | 51 | 312.236 |  |  |
|  | Total | 29359.956 | 63 |  |  |  |
| 5 | Regression | 13629.500 | 14 | 973.536 | 3.033 | .002f |
|  | Residual | 15730.457 | 49 | 321.030 |  |  |
|  | Total | 29359.956 | 63 |  |  |  |

1. Dependent Variable: MVPA_post
2. Predictors: (Constant), negaff_pre, MVPA_pre, AGwear_post, AGwear_pre, posaff_pre, needsupp_pre, Weight_pre, needsat_pre
3. Predictors: (Constant), negaff_pre, MVPA_pre, AGwear_post, AGwear_pre, posaff_pre, needsupp_pre, Weight_pre, needsat_pre, Age, Weight_post
4. Predictors: (Constant), negaff_pre, MVPA_pre, AGwear_post, AGwear_pre, posaff_pre, needsupp_pre, Weight_pre, needsat_pre, Age, Weight_post, needsupp_post
5. Predictors: (Constant), negaff_pre, MVPA_pre, AGwear_post, AGwear_pre, posaff_pre, needsupp_pre, Weight_pre, needsat_pre, Age, Weight_post, needsupp_post, needsat_post
6. Predictors: (Constant), negaff_pre, MVPA_pre, AGwear_post, AGwear_pre, posaff_pre,

## Coefficientsa

| Unstandardized Coefficients | | | | Standardized Coefficients |  |  | 95.0% Confidence Interval for B | | Correlations |
| --- | --- | --- | --- | --- | --- | --- | --- | --- | --- |
| Model |  | B | Std. Error | Beta | t | Sig. | Lower Bound | Upper Bound | Zero-order |
| 1 | (Constant) | 27.197 | 34.877 |  | .780 | .439 | -42.698 | 97.092 |  |
|  | AGwear_pre | -.005 | .021 | -.031 | -.233 | .816 | -.048 | .038 | .089 |
|  | AGwear_post | .013 | .017 | .097 | .772 | .444 | -.021 | .046 | .032 |
|  | MVPA_pre | .453 | .144 | .415 | 3.151 | .003 | .165 | .740 | .448 |
|  | Weight_pre | -.066 | .153 | -.058 | -.434 | .666 | -.373 | .240 | -.189 |
|  | needsupp_pre | 4.573 | 2.567 | .263 | 1.781 | .080 | -.572 | 9.717 | .284 |
|  | needsat_pre | -4.883 | 5.336 | -.138 | -.915 | .364 | -15.578 | 5.811 | .094 |
|  | posaff_pre | -1.274 | 4.882 | -.034 | -.261 | .795 | -11.057 | 8.510 | .102 |
|  | negaff_pre | -1.356 | 4.735 | -.039 | -.286 | .776 | -10.846 | 8.133 | -.076 |
| 2 | (Constant) | 39.440 | 33.713 |  | 1.170 | .247 | -28.180 | 107.061 |  |
|  | AGwear_pre | -.013 | .019 | -.083 | -.709 | .482 | -.051 | .024 | .089 |
|  | AGwear_post | .019 | .015 | .144 | 1.297 | .200 | -.010 | .049 | .032 |
|  | MVPA_pre | .339 | .129 | .311 | 2.623 | .011 | .080 | .598 | .448 |
|  | Weight_pre | 1.749 | .452 | 1.527 | 3.874 | <.001 | .844 | 2.655 | -.189 |
|  | needsupp_pre | 3.864 | 2.336 | .222 | 1.654 | .104 | -.821 | 8.549 | .284 |
|  | needsat_pre | .051 | 4.910 | .001 | .010 | .992 | -9.796 | 9.899 | .094 |
|  | posaff_pre | -3.905 | 4.363 | -.105 | -.895 | .375 | -12.657 | 4.847 | .102 |
|  | negaff_pre | 4.487 | 4.428 | .129 | 1.013 | .315 | -4.395 | 13.369 | -.076 |
|  | Age | -.011 | .323 | -.004 | -.033 | .974 | -.659 | .637 | .111 |
|  | Weight_post | -2.063 | .495 | -1.735 | -4.170 | <.001 | -3.055 | -1.071 | -.322 |
| 3 | (Constant) | 38.082 | 33.891 |  | 1.124 | .266 | -29.925 | 106.090 |  |
|  | AGwear_pre | -.015 | .019 | -.091 | -.773 | .443 | -.053 | .023 | .089 |

| Correlations | | | | Collinearity Statistics | |
| --- | --- | --- | --- | --- | --- |
| Model | | Partial | Part | Tolerance | VIF |
| 1 | (Constant) |  |  |  |  |
|  | AGwear_pre | -.031 | -.027 | .764 | 1.308 |
|  | AGwear_post | .103 | .090 | .862 | 1.161 |
|  | MVPA_pre | .391 | .366 | .776 | 1.288 |
|  | Weight_pre | -.058 | -.050 | .756 | 1.323 |
|  | needsupp_pre | .234 | .207 | .617 | 1.620 |
|  | needsat_pre | -.122 | -.106 | .596 | 1.679 |
|  | posaff_pre | -.035 | -.030 | .781 | 1.281 |
|  | negaff_pre | -.039 | -.033 | .731 | 1.368 |
| 2 | (Constant) |  |  |  |  |
|  | AGwear_pre | -.097 | -.072 | .755 | 1.324 |
|  | AGwear_post | .175 | .132 | .845 | 1.184 |
|  | MVPA_pre | .339 | .268 | .742 | 1.348 |
|  | Weight_pre | .470 | .395 | .067 | 14.922 |
|  | needsupp_pre | .222 | .169 | .576 | 1.736 |
|  | needsat_pre | .001 | .001 | .543 | 1.840 |
|  | posaff_pre | -.122 | -.091 | .755 | 1.325 |
|  | negaff_pre | .138 | .103 | .646 | 1.549 |
|  | Age | -.005 | -.003 | .827 | 1.209 |
|  | Weight_post | -.497 | -.426 | .060 | 16.617 |
| 3 | (Constant) |  |  |  |  |
|  | AGwear_pre | -.107 | -.079 | .749 | 1.335 |

| Unstandardized Coefficients | | | | Standardized Coefficients |  |  | 95.0% Confidence Interval for B | | Correlations |
| --- | --- | --- | --- | --- | --- | --- | --- | --- | --- |
| Model |  | B | Std. Error | Beta | t | Sig. | Lower Bound | Upper Bound | Zero-order |
|  | AGwear_post | .016 | .015 | .120 | 1.032 | .307 | -.015 | .047 | .032 |
|  | MVPA_pre | .357 | .132 | .328 | 2.709 | .009 | .093 | .622 | .448 |
|  | Weight_pre | 1.457 | .593 | 1.272 | 2.458 | .017 | .268 | 2.646 | -.189 |
|  | needsupp_pre | 2.682 | 2.806 | .154 | .956 | .344 | -2.949 | 8.312 | .284 |
|  | needsat_pre | -.092 | 4.932 | -.003 | -.019 | .985 | -9.989 | 9.806 | .094 |
|  | posaff_pre | -3.983 | 4.382 | -.107 | -.909 | .368 | -12.775 | 4.810 | .102 |
|  | negaff_pre | 4.574 | 4.447 | .131 | 1.029 | .308 | -4.350 | 13.497 | -.076 |
|  | Age | -.017 | .324 | -.006 | -.052 | .959 | -.668 | .634 | .111 |
|  | Weight_post | -1.784 | .616 | -1.500 | -2.896 | .006 | -3.020 | -.548 | -.322 |
|  | needsupp_post | 2.566 | 3.344 | .134 | .767 | .446 | -4.145 | 9.276 | .358 |
| 4 | (Constant) | 42.858 | 35.188 |  | 1.218 | .229 | -27.784 | 113.501 |  |
|  | AGwear_pre | -.013 | .019 | -.083 | -.689 | .494 | -.052 | .025 | .089 |
|  | AGwear_post | .016 | .016 | .119 | 1.021 | .312 | -.015 | .047 | .032 |
|  | MVPA_pre | .336 | .138 | .308 | 2.427 | .019 | .058 | .613 | .448 |
|  | Weight_pre | 1.579 | .636 | 1.378 | 2.483 | .016 | .302 | 2.856 | -.189 |
|  | needsupp_pre | 2.380 | 2.877 | .137 | .827 | .412 | -3.396 | 8.155 | .284 |
|  | needsat_pre | .708 | 5.171 | .020 | .137 | .892 | -9.672 | 11.089 | .094 |
|  | posaff_pre | -3.731 | 4.434 | -.100 | -.841 | .404 | -12.634 | 5.171 | .102 |
|  | negaff_pre | 4.831 | 4.501 | .139 | 1.073 | .288 | -4.205 | 13.866 | -.076 |
|  | Age | -.055 | .334 | -.019 | -.165 | .870 | -.725 | .615 | .111 |
|  | Weight_post | -1.904 | .657 | -1.601 | -2.898 | .006 | -3.224 | -.585 | -.322 |
|  | needsupp_post | 3.318 | 3.630 | .173 | .914 | .365 | -3.969 | 10.605 | .358 |
|  | needsat_post | -2.683 | 4.836 | -.085 | -.555 | .581 | -12.391 | 7.025 | .129 |
| 5 | (Constant) | 32.982 | 41.617 |  | .793 | .432 | -50.651 | 116.615 |  |
|  | AGwear_pre | -.011 | .020 | -.071 | -.573 | .569 | -.051 | .028 | .089 |
|  | AGwear_post | .018 | .016 | .132 | 1.100 | .277 | -.015 | .050 | .032 |

| Correlations | | | | Collinearity Statistics | |
| --- | --- | --- | --- | --- | --- |
| Model | | Partial | Part | Tolerance | VIF |
|  | AGwear_post | .142 | .106 | .781 | 1.281 |
|  | MVPA_pre | .352 | .278 | .717 | 1.394 |
|  | Weight_pre | .323 | .252 | .039 | 25.496 |
|  | needsupp_pre | .131 | .098 | .402 | 2.487 |
|  | needsat_pre | -.003 | -.002 | .543 | 1.843 |
|  | posaff_pre | -.125 | -.093 | .754 | 1.325 |
|  | negaff_pre | .141 | .105 | .645 | 1.550 |
|  | Age | -.007 | -.005 | .826 | 1.210 |
|  | Weight_post | -.373 | -.297 | .039 | 25.557 |
|  | needsupp_post | .106 | .079 | .346 | 2.892 |
| 4 | (Constant) |  |  |  |  |
|  | AGwear_pre | -.096 | -.071 | .737 | 1.358 |
|  | AGwear_post | .142 | .105 | .781 | 1.281 |
|  | MVPA_pre | .322 | .250 | .661 | 1.513 |
|  | Weight_pre | .328 | .256 | .035 | 28.979 |
|  | needsupp_pre | .115 | .085 | .388 | 2.579 |
|  | needsat_pre | .019 | .014 | .500 | 1.998 |
|  | posaff_pre | -.117 | -.087 | .747 | 1.339 |
|  | negaff_pre | .149 | .111 | .638 | 1.567 |
|  | Age | -.023 | -.017 | .791 | 1.264 |
|  | Weight_post | -.376 | -.299 | .035 | 28.703 |
|  | needsupp_post | .127 | .094 | .297 | 3.362 |
|  | needsat_post | -.077 | -.057 | .450 | 2.221 |
| 5 | (Constant) |  |  |  |  |
|  | AGwear_pre | -.082 | -.060 | .722 | 1.385 |
|  | AGwear_post | .155 | .115 | .760 | 1.316 |

| Unstandardized Coefficients | | | | Standardized Coefficients |  |  | 95.0% Confidence Interval for B | | Correlations |
| --- | --- | --- | --- | --- | --- | --- | --- | --- | --- |
| Model |  | B | Std. Error | Beta | t | Sig. | Lower Bound | Upper Bound | Zero-order |
|  | MVPA_pre | .353 | .142 | .323 | 2.481 | .017 | .067 | .638 | .448 |
|  | Weight_pre | 1.585 | .649 | 1.384 | 2.443 | .018 | .281 | 2.889 | -.189 |
|  | needsupp_pre | 1.947 | 2.971 | .112 | .655 | .515 | -4.024 | 7.919 | .284 |
|  | needsat_pre | .646 | 5.256 | .018 | .123 | .903 | -9.916 | 11.208 | .094 |
|  | posaff_pre | -2.843 | 5.084 | -.077 | -.559 | .579 | -13.060 | 7.373 | .102 |
|  | negaff_pre | 3.531 | 6.163 | .101 | .573 | .569 | -8.854 | 15.916 | -.076 |
|  | Age | .014 | .363 | .005 | .037 | .970 | -.715 | .742 | .111 |
|  | Weight_post | -1.921 | .672 | -1.615 | -2.859 | .006 | -3.270 | -.571 | -.322 |
|  | needsupp_post | 4.199 | 3.909 | .219 | 1.074 | .288 | -3.655 | 12.054 | .358 |
|  | needsat_post | -.934 | 5.399 | -.030 | -.173 | .863 | -11.784 | 9.915 | .129 |
|  | posaff_post | -3.200 | 5.176 | -.094 | -.618 | .539 | -13.602 | 7.201 | .164 |
|  | negaff_post | 3.662 | 8.145 | .082 | .450 | .655 | -12.706 | 20.031 | -.170 |

| Correlations | | | | Collinearity Statistics | |
| --- | --- | --- | --- | --- | --- |
| Model | | Partial | Part | Tolerance | VIF |
|  | MVPA_pre | .334 | .259 | .643 | 1.555 |
|  | Weight_pre | .330 | .255 | .034 | 29.332 |
|  | needsupp_pre | .093 | .069 | .374 | 2.676 |
|  | needsat_pre | .018 | .013 | .498 | 2.008 |
|  | posaff_pre | -.080 | -.058 | .584 | 1.712 |
|  | negaff_pre | .082 | .060 | .350 | 2.857 |
|  | Age | .005 | .004 | .689 | 1.452 |
|  | Weight_post | -.378 | -.299 | .034 | 29.169 |
|  | needsupp_post | .152 | .112 | .264 | 3.792 |
|  | needsat_post | -.025 | -.018 | .371 | 2.693 |
|  | posaff_post | -.088 | -.065 | .469 | 2.133 |
|  | negaff_post | .064 | .047 | .326 | 3.067 |

1. Dependent Variable: MVPA_post

## Excluded Variablesa

| Model Beta In | | | t | Sig. | Partial Correlation | Collinearity Statistics | | |
| --- | --- | --- | --- | --- | --- | --- | --- | --- |
|  |  |  |  |  |  | Tolerance | VIF | Minimum Tolerance |
| 1 | Age | .099b | .792 | .432 | .107 | .869 | 1.151 | .585 |
|  | Weight_post | -1.732b | -4.307 | <.001 | -.506 | .063 | 15.817 | .063 |
|  | needsupp_post | .441b | 2.999 | .004 | .378 | .544 | 1.838 | .462 |
|  | needsat_post | .247b | 1.905 | .062 | .251 | .764 | 1.308 | .591 |
|  | posaff_post | .161b | 1.189 | .239 | .160 | .727 | 1.375 | .594 |
|  | negaff_post | -.174b | -1.079 | .285 | -.145 | .514 | 1.945 | .442 |
| 2 | needsupp_post | .134c | .767 | .446 | .106 | .346 | 2.892 | .039 |
|  | needsat_post | -.033c | -.230 | .819 | -.032 | .523 | 1.911 | .041 |
|  | posaff_post | -.074c | -.555 | .581 | -.077 | .590 | 1.695 | .049 |
|  | negaff_post | .038c | .237 | .814 | .033 | .415 | 2.412 | .055 |
| 3 | needsat_post | -.085d | -.555 | .581 | -.077 | .450 | 2.221 | .035 |
|  | posaff_post | -.112d | -.804 | .425 | -.112 | .543 | 1.842 | .037 |
|  | negaff_post | .104d | .596 | .554 | .083 | .347 | 2.885 | .039 |
| 4 | posaff_post | -.097e | -.638 | .526 | -.090 | .469 | 2.131 | .034 |
|  | negaff_post | .086e | .473 | .639 | .067 | .326 | 3.064 | .034 |

1. Dependent Variable: MVPA_post
2. Predictors in the Model: (Constant), negaff_pre, MVPA_pre, AGwear_post, AGwear_pre, posaff_pre, needsupp_pre,

Weight_pre, needsat_pre

1. Predictors in the Model: (Constant), negaff_pre, MVPA_pre, AGwear_post, AGwear_pre, posaff_pre, needsupp_pre,

Weight_pre, needsat_pre, Age, Weight_post

1. Predictors in the Model: (Constant), negaff_pre, MVPA_pre, AGwear_post, AGwear_pre, posaff_pre, needsupp_pre,

Weight_pre, needsat_pre, Age, Weight_post, needsupp_post

1. Predictors in the Model: (Constant), negaff_pre, MVPA_pre, AGwear_post, AGwear_pre, posaff_pre, needsupp_pre, Weight_pre, needsat_pre, Age, Weight_post, needsupp_post, needsat_post

Model Dimension Eigenvalue

Condition Index

(Constant)

AGwear_pre

AGwear_post

MVPA_pre

Weight_pre

needsupp_pre

1 1 8.522

2 .211

3 .134

4 .040

5 .034

6 .024

7 .018

8 .015

9 .004

2 1 10.463

2 .227

3 .134

4 .049

5 .037

6 .033

7 .024

8 .016

9 .013

10 .004

11 .001

3 1 11.430

2 .228

3 .143

4 .050

5 .039

6 .034

1.000

6.353

7.985

14.581

15.896

18.952

22.058

24.213

45.647

1.000

6.787

8.844

14.625

16.771

17.853

20.906

25.669

28.293

53.549

108.921

1.000

7.081

8.936

15.181

17.048

18.359

.00

.00

.00

.00

.00

.00

.00

.02

.97

.00

.00

.00

.00

.00

.00

.00

.00

.01

.97

.01

.00

.00

.00

.00

.00

.00

.00

.00

.00

.06

.09

.04

.00

.39

.42

.00

.00

.00

.04

.06

.05

.00

.09

.44

.32

.01

.00

.00

.00

.03

.07

.01

.00

.00

.00

.25

.00

.00

.01

.72

.02

.00

.00

.00

.09

.00

.10

.20

.30

.27

.01

.01

.00

.00

.00

.08

.00

.09

.00

.67

.07

.03

.01

.19

.01

.00

.01

.00

.58

.08

.02

.14

.15

.00

.00

.00

.01

.02

.00

.57

.06

.02

.05

.24

.00

.01

.00

.01

.03

.53

.08

.05

.30

.00

.00

.00

.00

.01

.00

.00

.00

.00

.01

.97

.00

.00

.00

.00

.00

.00

.00

.00

.03

.21

.25

.03

.34

.12

.02

.00

.00

.03

.10

.32

.00

.01

.09

.43

.00

.02

.00

.00

.02

.08

.12

.04

| Model Dimension | | needsat_pre | posaff_pre | negaff_pre | Age | Weight_post | needsupp_post | needsat_post | posaff_post | negaff_post |
| --- | --- | --- | --- | --- | --- | --- | --- | --- | --- | --- |
| 1 | 1 | .00 | .00 | .00 |  |  |  |  |  |  |
|  | 2 | .00 | .00 | .02 |  |  |  |  |  |  |
|  | 3 | .01 | .01 | .41 |  |  |  |  |  |  |
|  | 4 | .03 | .00 | .18 |  |  |  |  |  |  |
|  | 5 | .00 | .47 | .00 |  |  |  |  |  |  |
|  | 6 | .00 | .31 | .19 |  |  |  |  |  |  |
|  | 7 | .76 | .11 | .02 |  |  |  |  |  |  |
|  | 8 | .02 | .05 | .16 |  |  |  |  |  |  |
|  | 9 | .16 | .05 | .02 |  |  |  |  |  |  |
| 2 | 1 | .00 | .00 | .00 | .00 | .00 |  |  |  |  |
|  | 2 | .00 | .00 | .01 | .00 | .00 |  |  |  |  |
|  | 3 | .01 | .01 | .36 | .00 | .00 |  |  |  |  |
|  | 4 | .01 | .00 | .03 | .11 | .00 |  |  |  |  |
|  | 5 | .00 | .06 | .16 | .01 | .01 |  |  |  |  |
|  | 6 | .03 | .37 | .18 | .05 | .00 |  |  |  |  |
|  | 7 | .09 | .37 | .03 | .31 | .00 |  |  |  |  |
|  | 8 | .55 | .09 | .12 | .08 | .00 |  |  |  |  |
|  | 9 | .15 | .05 | .02 | .24 | .00 |  |  |  |  |
|  | 10 | .08 | .03 | .01 | .15 | .00 |  |  |  |  |
|  | 11 | .07 | .02 | .08 | .05 | .98 |  |  |  |  |
| 3 | 1 | .00 | .00 | .00 | .00 | .00 | .00 |  |  |  |
|  | 2 | .00 | .00 | .01 | .00 | .00 | .00 |  |  |  |
|  | 3 | .01 | .01 | .33 | .00 | .00 | .01 |  |  |  |
|  | 4 | .00 | .01 | .04 | .12 | .00 | .01 |  |  |  |
|  | 5 | .01 | .14 | .09 | .01 | .00 | .03 |  |  |  |
|  | 6 | .06 | .20 | .25 | .02 | .00 | .02 |  |  |  |

Model Dimension Eigenvalue

Condition Index

(Constant)

AGwear_pre

AGwear_post

MVPA_pre

Weight_pre

needsupp_pre

7 .025

8 .022

9 .014

10 .011

11 .004

12 .001

4 1 12.403

2 .233

3 .147

4 .050

5 .040

6 .035

7 .027

8 .024

9 .017

10 .014

11 .007

12 .003

13 .000

5 1 14.278

2 .241

3 .203

4 .059

5 .048

6 .039

7 .036

21.469

22.985

28.077

32.319

56.117

146.341

1.000

7.302

9.198

15.789

17.653

18.899

21.258

22.972

26.955

29.255

41.754

59.661

162.050

1.000

7.691

8.396

15.495

17.285

19.157

19.995

.00

.00

.01

.00

.97

.01

.00

.00

.00

.00

.00

.00

.00

.00

.00

.01

.01

.94

.04

.00

.00

.00

.00

.00

.00

.00

.01

.03

.44

.09

.33

.00

.00

.00

.00

.03

.07

.00

.03

.00

.04

.42

.16

.25

.00

.00

.00

.00

.01

.00

.08

.02

.06

.20

.40

.08

.01

.06

.00

.00

.00

.08

.00

.06

.03

.29

.00

.39

.07

.02

.05

.00

.00

.00

.02

.04

.00

.06

.00

.00

.00

.00

.01

.05

.00

.53

.03

.03

.02

.27

.00

.00

.00

.00

.01

.01

.09

.00

.39

.15

.01

.08

.10

.05

.00

.00

.00

.00

.01

.98

.00

.00

.00

.00

.00

.00

.00

.00

.00

.00

.00

.01

.98

.00

.00

.00

.00

.00

.00

.00

.00

.01

.00

.66

.00

.07

.00

.00

.02

.07

.07

.09

.05

.02

.12

.00

.50

.03

.03

.00

.00

.00

.08

.02

.13

.00

| Model Dimension | | needsat_pre | posaff_pre | negaff_pre | Age | Weight_post | needsupp_post | needsat_post | posaff_post | negaff_post |
| --- | --- | --- | --- | --- | --- | --- | --- | --- | --- | --- |
|  | 7 | .18 | .46 | .04 | .10 | .00 | .07 |  |  |  |
|  | 8 | .07 | .01 | .00 | .34 | .00 | .19 |  |  |  |
|  | 9 | .13 | .14 | .16 | .01 | .00 | .05 |  |  |  |
|  | 10 | .42 | .00 | .03 | .22 | .00 | .22 |  |  |  |
|  | 11 | .07 | .03 | .00 | .16 | .00 | .00 |  |  |  |
|  | 12 | .05 | .01 | .04 | .02 | .99 | .41 |  |  |  |
| 4 | 1 | .00 | .00 | .00 | .00 | .00 | .00 | .00 |  |  |
|  | 2 | .00 | .00 | .01 | .00 | .00 | .00 | .00 |  |  |
|  | 3 | .01 | .01 | .33 | .00 | .00 | .01 | .00 |  |  |
|  | 4 | .00 | .01 | .04 | .12 | .00 | .01 | .00 |  |  |
|  | 5 | .02 | .15 | .07 | .01 | .00 | .04 | .01 |  |  |
|  | 6 | .07 | .12 | .24 | .02 | .00 | .01 | .02 |  |  |
|  | 7 | .07 | .27 | .06 | .00 | .00 | .06 | .11 |  |  |
|  | 8 | .03 | .25 | .02 | .31 | .00 | .01 | .03 |  |  |
|  | 9 | .26 | .01 | .01 | .23 | .00 | .07 | .20 |  |  |
|  | 10 | .15 | .14 | .17 | .00 | .00 | .04 | .00 |  |  |
|  | 11 | .28 | .02 | .02 | .08 | .00 | .53 | .44 |  |  |
|  | 12 | .03 | .02 | .00 | .19 | .00 | .06 | .06 |  |  |
|  | 13 | .09 | .00 | .05 | .04 | .99 | .18 | .12 |  |  |
| 5 | 1 | .00 | .00 | .00 | .00 | .00 | .00 | .00 | .00 | .00 |
|  | 2 | .00 | .00 | .02 | .00 | .00 | .00 | .00 | .00 | .01 |
|  | 3 | .00 | .00 | .06 | .00 | .00 | .00 | .00 | .00 | .04 |
|  | 4 | .03 | .01 | .05 | .03 | .00 | .00 | .00 | .02 | .11 |
|  | 5 | .01 | .07 | .08 | .06 | .00 | .03 | .01 | .00 | .05 |
|  | 6 | .00 | .07 | .03 | .00 | .00 | .01 | .00 | .01 | .00 |
|  | 7 | .01 | .15 | .05 | .02 | .00 | .01 | .01 | .09 | .01 |

Model Dimension Eigenvalue Condition Index (Constant) AGwear_pre AGwear_post MVPA_pre Weight_pre needsupp_pre

8 .029 22.322 .00 .02 .17 .06 .00 .00

9 .019 27.461 .00 .04 .22 .01 .00 .09

10 .016 29.431 .00 .00 .16 .00 .00 .02

11 .013 33.108 .00 .49 .17 .01 .00 .00

12 .010 38.598 .00 .00 .01 .01 .00 .14

13 .007 45.411 .00 .09 .07 .01 .00 .48

14 .003 74.175 .93 .24 .02 .03 .01 .00

15 .000 175.454 .06 .00 .06 .09 .98 .03

## Collinearity Diagnosticsa

Variance Proportions

| Model Dimension | | needsat_pre | posaff_pre | negaff_pre | Age | Weight_post | needsupp_post | needsat_post | posaff_post | negaff_post |
| --- | --- | --- | --- | --- | --- | --- | --- | --- | --- | --- |
|  | 8 | .09 | .00 | .16 | .21 | .00 | .00 | .00 | .02 | .09 |
|  | 9 | .04 | .17 | .17 | .00 | .00 | .01 | .15 | .02 | .11 |
|  | 10 | .42 | .01 | .18 | .20 | .00 | .06 | .01 | .00 | .08 |
|  | 11 | .01 | .26 | .02 | .08 | .00 | .10 | .01 | .00 | .17 |
|  | 12 | .00 | .26 | .02 | .01 | .00 | .09 | .21 | .81 | .00 |
|  | 13 | .27 | .00 | .00 | .06 | .00 | .54 | .41 | .01 | .02 |
|  | 14 | .04 | .00 | .09 | .28 | .00 | .00 | .08 | .00 | .29 |
|  | 15 | .08 | .00 | .06 | .05 | .99 | .17 | .10 | .01 | .01 |

1. Dependent Variable: MVPA_post

## Residuals Statisticsa

| Minimum | | Maximum | Mean | Std. Deviation | N |
| --- | --- | --- | --- | --- | --- |
| Predicted Value | 12.53 | 91.55 | 45.67 | 14.709 | 64 |
| Std. Predicted Value | -2.253 | 3.119 | .000 | 1.000 | 64 |
| Standard Error of Predicted Value | 5.116 | 13.117 | 8.500 | 1.744 | 64 |
| Adjusted Predicted Value | 7.56 | 105.23 | 46.36 | 16.039 | 64 |
| Residual | -32.024 | 40.495 | .000 | 15.802 | 64 |
| Std. Residual | -1.787 | 2.260 | .000 | .882 | 64 |
| Stud. Residual | -2.026 | 2.436 | -.016 | .990 | 64 |
| Deleted Residual | -41.151 | 47.035 | -.694 | 20.079 | 64 |
| Stud. Deleted Residual | -2.095 | 2.572 | -.014 | 1.007 | 64 |
| Mahal. Distance | 4.153 | 32.780 | 13.781 | 6.096 | 64 |
| Cook's Distance | .000 | .082 | .018 | .023 | 64 |
| Centered Leverage Value | .066 | .520 | .219 | .097 | 64 |

1. Dependent Variable: MVPA_post

**Charts**

# Normal P-P Plot of Regression Standardized Residual Dependent Variable: MVPA_post

1.0

0.8

**Expected Cum Prob**

0.6

0.4

0.2

0.0

0.0

0.2

0.4

0.6

0.8

1.0

# Observed Cum Prob

**Scatterplot Dependent Variable: MVPA_post**

3

**Regression Standardized Residual**

2

1

0

-1

-2

-2 0 2 4

**Regression Standardized Predicted Value**
